# Influence of Conditioned Media on the Re-Differentiation Capacity of Human Chondrocytes in 3D Spheroid Cultures

**DOI:** 10.3390/jcm9092798

**Published:** 2020-08-30

**Authors:** Annett Klinder, Sophie Kussauer, Bettina Hiemer, Andreas Wree, Rainer Bader, Anika Jonitz-Heincke

**Affiliations:** 1 Department of Orthopedics, Biomechanics and Implant Technology Research Laboratory, Rostock University Medical Center, 18057 Rostock, Germany; sophie.kussauer@med.uni-rostock.de (S.K.); bettina.hiemer@med.uni-rostock.de (B.H.); rainer.bader@med.uni-rostock.de (R.B.); anika.jonitz-heincke@med.uni-rostock.de (A.J.-H.); 2 Department of Anatomy, Rostock University Medical Center, 18057 Rostock, Germany; andreas.wree@med.uni-rostock.de

**Keywords:** hyaline cartilage, spheroids, medium conditioning, re-differentiation, extracellular matrix

## Abstract

A major challenge of cell-based therapy for cartilage lesions is the preservation of the chondrogenic phenotype during ex vivo cell cultivation. In this in vitro study, the chondro-inductive capacity of two different hyaline cartilage-conditioned cell culture media on human chondrocytes in 3D spheroids was determined. Media were conditioned by incubation of 200 mg/mL vital or devitalized cartilage matrix in growth media over 35 days. The media were analyzed for the content of soluble procollagen type (Col) II and glycosaminoglycans (GAGs) as well as released TGF-β1, IGF-1 and IGFBP3. Unconditioned medium served as a negative control while the positive medium control was supplemented with TGF-β1 and IGF-1. Spheroid cultures prepared from human chondrocytes were cultivated at 37 °C, 5% CO_2_ and 21% O_2_ in the respective media and controls. After 14 and 35 days, the deposition of ECM components was evaluated by histological analysis. Vital cartilage-conditioned medium contained significantly higher levels of Col II and active TGF-β1 compared to medium conditioned with the devitalized cartilage matrix. Despite these differences, the incubation with vital as well as devitalized cartilage conditioned medium led to similar results in terms of deposition of proteoglycans and collagen type II, which was used as an indicator of re-differentiation of human chondrocytes in spheroid cultures. However, high density 3D cell cultivation showed a positive influence on re-differentiation.

## 1. Introduction

Defects of hyaline cartilage have recently been identified as the majority of diseases of the musculoskeletal system in both humans and animals [1]. The spectrum of disease extends from traumata in mainly young athletes to age-related degenerative processes which are characterized by chondral or osteochondral lesions [2]. The defects lead to a decline in load-bearing capacity and thus a change in biomechanical properties of cartilage. Due to its lack of vascularization and the dominance of a post-mitotic cell type, cartilage tissue has little potential for the regeneration of defects and the maintenance of the tissue. Defect regeneration leads often to fibrocartilage-like tissue in patients [1]. This replacement tissue is characterized by a high amount of collagen type I and low proteoglycan content and lacks important properties such as resistance to biophysical loading. Untreated defects can result in an ongoing loss of tissue function which may end in osteoarthritis [1,3]. Over the years, some surgical treatment procedures have emerged including arthroscopic debridement and lavage, subchondral bone microfracture, osteochondral transplantation and autologous chondrocyte implantation (ACI). However, these treatments can lack in restoration of functional properties of the replaced cartilaginous tissue [4]. In case of ACI, a disadvantage is the de-differentiation of chondrocytic cells during in vitro expansion. The loss of the chondrogenic phenotype already after the first passage in vitro is well documented [5,6] and is characterized by morphological changes, a decrease in secretion of cartilage-specific matrix proteins such as collagen II and aggrecan and the up-regulation of mesenchymal stem cell genes [6,7,8,9,10,11,12,13,14].

Indeed, our group showed that the surface markers of mesenchymal cells, cluster of differentiation (CD)166, CD105, CD44 and CD29, changed incrementally from passage 0 to passage 4 during in vitro cultivation of isolated human chondrocytes in monolayer cultures [15]. Therefore, research has focused on the optimization of chondrocyte re-differentiation by using essential chondrogenic growth factors, improved three-dimensional culture conditions or physiologically approximated oxygen partial pressures [15,16,17,18,19]. While re-differentiation of chondrocytes was already described after cultivation in various 3D systems [20] we were only able to detect immunostained collagen type II and aggrecan proteins in alginate bead culture after stimulation with chondrogenic growth factors insulin like growth factor (IGF-)1 (50 ng/mL) and transforming growth factor (TGF-) β1 (50 ng/mL) [15]. The stimulatory concentration of IGF-1 was similar to that reported for cartilage in the literature [21]. However, whereas IGF-1 showed beneficial effects in articular cartilage defects [22] high systemic concentrations have been linked to increased cancer risk [23]. Contrary to IGF-1, the concentration of 50 ng/mL for TGF-β1 far exceeds the concentration of active TGF-β1 in articular cartilage [24]. Induction of proteoglycan synthesis with exogenous TGF-β1 reached saturation already at approximately 5 ng/mL [25] and high concentrations of TGF-β1 are thought to activate the pSMAD1/5 signaling pathway which is associated with hypertrophy in chondrocytes and leads to their terminal differentiation [26]. We also showed that the utilization of the high concentrations of growth factors enhanced the production of collagen type I over the production of collagen type II [27], thus favoring the formation of fibrous cartilage. In order to overcome the disadvantages associated with high concentrations of single growth factors, investigations have been made concerning the impact of conditioned medium as this might contain co-stimulatory factors such as extracellular matrix (ECM) components apart from the more physiological concentrations of growth factors. For example, bone marrow mesenchymal stem cells (BM-MSC) have been shown to upregulate collagen type II expression and form neocartilage after cultivation with articular chondrocyte conditioned medium supplemented with and without TFG-β onto fibrous collagen scaffolds [28]. Furthermore, conditioned medium derived from combined chondrocyte/scaffold constructs without addition of further growth factors led to the formation of cartilaginous tissue and expression of chondrogenic marker genes in cultivated BM-MSCs [29,30]. Moreover, conditioned medium from human adipose derived mesenchymal stem cell (AD-MSC) was shown to decrease the production of oxidative stress and proinflammatory cytokines and protect chondrocytes against degeneration by releasing paracrine factors [31]. In conclusion, conditioned medium was shown to increase chondrocyte differentiation [29] and protect cells from oxidative stress [32]. There is also some evidence of chondroinductive effects of the native cartilage matrix that supports the rationale behind the generation of the conditioned medium from cartilage. For example, the presence of porcine decellularized native cartilage or cell derived matrix was shown to have positive effects on human chondrocyte gene expression in vitro by increasing the expression of collagen II and aggrecan [33]. Moreover, cultivation of chondrocytes on inactivated cartilage matrix without supplementation of additional growth factors showed re-differentiation by expression of cartilage specific genes. Hence, an inactivated cartilage matrix seems to drive chondrocyte differentiation [34]. However, little is known about the mechanisms by which the cartilage matrix promotes chondrogenesis [33]. Induction of chondrogenesis could result either from direct transmission of important factors by cell matrix contact or the release of factors into the surrounding area. Hence, the aim of the present study was to identify the re-differentiation capacity of human chondrocytes in spheroid cultures by using conditioned media derived from devitalized or vital cartilage. Hiemer et al. [34] analyzed the potential of human cartilage devitalized by high hydrostatic pressure treatment as scaffolds. They showed an effect of promoting re-differentiation in dedifferentiated chondrocytes by culture on the devitalized cartilage. Direct (cell-to-matrix interaction) or indirect (effects of growth factor related mediators) induction of chondrogenesis might be responsible for the effects driving re-differentiation. Therefore, the focus of our study was to gain insight into the question whether (i) the observed differentiation-driving effects originate from factors being released by the cartilage matrix or (ii) if there is a continued need for vital cells within the cartilage matrix in order to synthesize mediators which contribute to a chondrogenesis promoting environment.

## 2. Experimental Section

### 2.1. Cartilage Harvest

Hyaline cartilage was extracted from articular cartilage donated from patients (*n* = 8; 2 females: 60 years (range 48–72 years); 6 males: 62 years (range 44–88 years)) undergoing primary knee replacement. Samples were collected with patient informed consent. The procedures were approved by the Local Ethical Committee of the University of Rostock (registration number: A2009 17). Cartilage tissue was used either for generating conditioned medium or for isolating chondrocytes as described below.

### 2.2. Generation of Conditioned Medium

**Figure 1:**
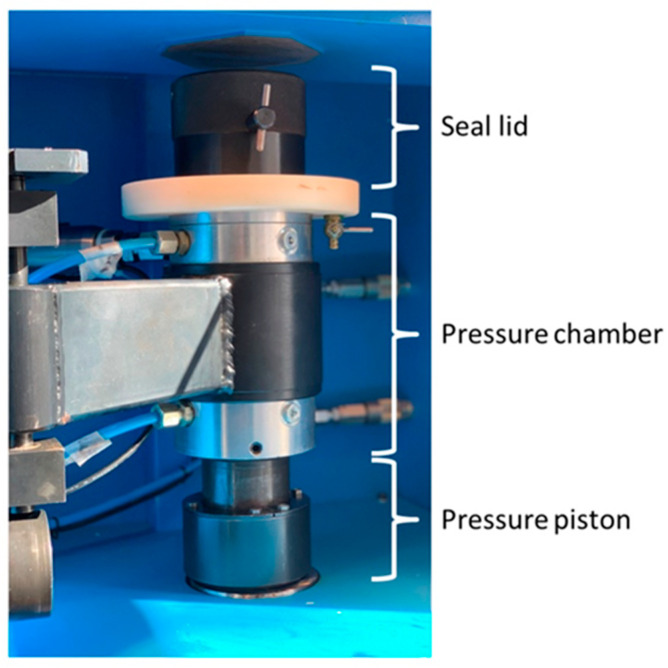
Detail view of the high hydrostatic pressure reactor: Cryo-tubes with cartilage were placed in the pressure chamber.

Cartilage tissue was washed with phosphate buffered saline (PBS, PAA, Coelbe, Germany), weighed, and finally cut into 2 × 2 mm slices. Cartilage samples were split into two groups: (i) vital cartilage (VC) or (ii) high hydrostatic pressure treated cartilage (HHPTC). VC was left untreated, whereas HHPTC was treated with high hydrostatic pressure using a specific reactor (HDR-100, Retrofit GmbH, Ditzingen, Germany) (Figure 1) according to previous description of Hiemer et al. [34]. Briefly, HHPTC samples were placed in cryo-tubes completely filled with Dulbecco’s Modified Eagle Medium (DMEM, Invitrogen, Darmstadt, Germany) and transferred to the glycol-filled pressure chamber of the high hydrostatic pressure device. After a pressure built-up phase (34.3 MPa/min) the sample was treated for 10 min with 480 MPa at 20 °C.

**Figure 2:**
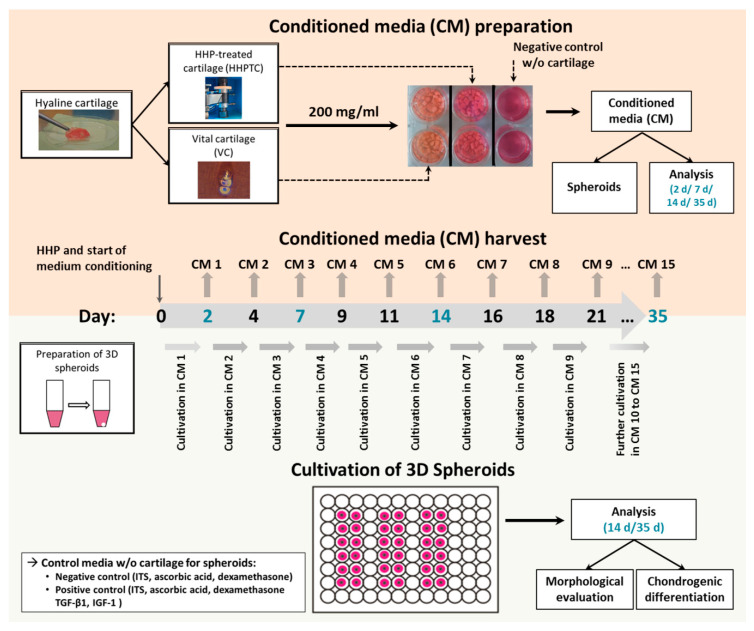
Flow diagram of experimental approach and methods. The upper panel (light orange) shows the preparation of conditioned medium of HHP-treated and vital hyaline cartilage tissue. The tissue slices were cultivated for a total period of 35 days. Medium was changed and harvested every two to three days for subsequent use for 3D spheroid cultures. In addition, the release of soluble chondrogenic mediators was analyzed on day 2 (conditioned medium (CM 1), day 7 (CM 3), day 14 (CM 6) and day 35 (CM 15). The lower panel (light green) depicts the preparation and cultivation of chondrocytic spheroid cultures. After a two-day incubation in basal medium (negative control medium), spheroids were supplied with the respective CM of vital and HHP-treated cartilage. Spheroids, cultivated with basal medium containing ITS™, ascorbic acid and dexamethasone (negative control) or basal medium plus chondrogenic growth factors TGF-β1 and IGF-1 (positive control) served as controls. After 14 d and 35 d of incubation, spheroids were analyzed regarding morphological changes and chondrogenic differentiation.

For up to 35 days VC and HHPTC (200 mg tissue/mL) were kept in DMEM supplemented with 1% penicillin/streptomycin (Gibco, Carlsbad, CA, USA), 1% fungicide (PAA, Coelbe, Germany) 1% insulin-transferrin-sodium selenite media supplement (ITS™, Corning, Bedford, MA, USA), ascorbic acid (final concentration 50 ng/mL; Sigma-Aldrich, Munich, Germany) and dexamethasone (final concentration 100 nM; Sigma-Aldrich, Munich, Germany), further referred to as chondrocyte cultivation medium at 37 °C and 5% CO_2_. Every two to four days of incubation the medium was replaced. The harvested conditioned medium was subjected to sterile filtration (0.22 µm filter, Rotilabo-syringe filters, Carl Roth GmbH + Co. KG, Karlsruhe, Germany) and stored at −20 °C. Conditioned media of vital, untreated cartilage (VC) and of pressure treated cartilage (HHPTC) were used for cultivation of human chondrocytes for induction of chondrogenic differentiation (Figure 2).

### 2.3. Cell Viability Analysis

To assess the devitalization efficiency of HHP-treated cartilage, live/dead staining was performed with the “Live/Dead^®^ Assay” (Molecular Probes, Life technologies, Carlsbad, CA, USA), containing the green fluorescent Calcein-acetoxymethyl as the “Live“ dye and red fluorescent Ethidium homodimer-1 as the “Dead” dye, as described in the manufacturer’s instruction. Briefly, after completion of the conditioning of the media at day 35, the remaining cartilage (vital and pressure treated) was digested with trypsin and collagenase as described in Chapter 2.6 in a total volume of 1 mL per sample. Following digestion 0.25 mL of the solution was analyzed by Trypan blue staining in a Thoma haemocytometer while 0.75 mL of the solution were seeded into a 6-well plate and cultured with chondrocyte cultivation medium for 8 days. At day 8 the medium was removed, the cells were washed with PBS and covered with the dye working solution (20 µL of 2 mM Ethidium homodimer-1 solution and 5 µL of 4 mM Calcein AM solution in 10 mL of sterile PBS). After an incubation time of 30 min at room temperature in the dark the assay was analyzed under the fluorescence microscope (Nikon Type 120 with the Nikon digital sight camera). The software “NIS elements 3.2” was used. Dead cells appeared red and the live cells appeared green.

### 2.4. Determination of Cell Count in Spheroid Cultures

For cell proliferation analysis the “CyQUANT^®^ NF Cell Proliferation assay kit” (Thermo Fisher Scientific, Waltham, MA, USA) was used and performed according to manufacturer’s instructions. The assay is based on a DNA-binding dye. For the standard curve 100,000 cells in 50 µL of 1× HBSS buffer (Hank’s Balanced Salt Solution, Thermo Fisher Scientific, Waltham, MA, USA) were diluted 1:2 in five consecutive steps. Next a dilution series of the digested spheroids with 1x HBSS buffer was prepared. Then 50 µL of each sample were transferred into a 96-well plate. For both the standard curve and the dilution series, 1 µL of dye reagent was mixed with 250 µL 1x HBSS buffer for each sample. After adding 50 µL of dye binding solution to each well, the plate was incubated for 1 h at 37 °C covered with a light-tight foil. Finally, the CyQuant^®^ NF-Assay was measured with a spectrophotometer (Tecan-reader Infinite 200 Pro (Tecan group Ltd., Maennedorf, Switzerland) with 485 nm for excitation and 535 nm for emission.

### 2.5. Analysis of Cartilage Conditioned Media

Analysis of newly synthesized ECM components such as collagen II and sulphated glycosaminoglycans was performed in order to determine re-differentiation of chondrocytes. For the analysis of type II procollagen concentration, the ProCollagen II C-propeptide Assay (IBEX Pharmaceuticals, Montreal, QC, Canada) was used. Absorbances were measured in a spectrophotometer (Dynex Opsys MR 96, Dynex Technologies, Denkendorf, Germany).

Sulphated proteoglycans and glycosaminoglycans (GAGs) were quantified following the manufacturer’s instructions of the BlyScan™ sulphated glycosaminoglycan (GAG) assay (Biocolor, Carrickfergus, UK). 

IGF-1 protein in conditioned media was detected with the Human IGF1 SimpleStep ^®^ ELISA Kit (Abcam, Eugene, OR, USA). Additionally, the release of IGF-binding protein (IGFBP)3 was quantified using the Human IGFBP3 ELISA Kit (Abcam, Eugene, OR, USA). The LEGEND MAX™ Free Active TGF-β1 ELISA Kit (Biolegend, San Diego, CA, USA) was performed in order to quantify free active TGF-β1 protein. All enzyme-linked immunosorbent assays (ELISA) based kits were performed according to manufacturer’s instructions and analyzed with a reproducible standard curve using a spectrophotometer (Tecan reader Infinite 200 Pro, Tecan group Ltd. Maennedorf, Switzerland). All protein data were normalized to the total protein concentration. The Qubit^®^ protein assay kit (ThermoFisher Scientific, Waltham, MA, USA) was used for the determination of protein concentration according to the manufacturer’s instructions. 

### 2.6. Isolation and Cultivation of Chondrocytes in 3D Spheroid Cultures

Isolation of chondrocytes was performed as described by Jonitz et al. [15]. Briefly, cartilage was cut into small pieces, washed with PBS and subsequently treated at 37 °C under constant agitation with trypsin for 20 min followed by collagenase A treatment overnight. Isolated chondrocytes were cultivated in DMEM supplemented with 10% fetal calf serum (FCS, PAN Biotech GmbH, Aidenbach, Germany), 1% penicillin/streptomycin, 1% fungicide and ascorbic acid (50 ng/mL) over two passages. In passage 3 cells were harvested to create 3D spheroids by subsequently centrifuging 5 × 10^5^ cells in 0.5 mL at 118× *g* for 5 min. Spheroids were incubated at 37 °C, 5% CO_2_ and 21% O_2_ in chondrocyte cultivation medium for three days. Afterwards, the spheroids were transferred into a 96 well suspension plate (Greiner BioOne, Frickenhausen, Germany) and cultured under the conditions mentioned in Table 1.

Prior to the re-differentiation experiments, the best conditions for conditioned medium generation and storage were determined. The conditioned media were stored at −20 °C. The defrosted conditioned media (VC conditioned medium, HHPTC conditioned medium, control) was mixed 1:1 with fresh chondrocyte cultivation medium to guarantee a sufficient supply with essential nutrients for the cultivated chondrocytes in 3D spheroid cultures. Preliminary investigations in monolayer cultures showed that cultivation in conditioned media from vital cartilage promoted the growth of chondrocytes compared to chondrocyte cultivation medium (NC) with the best results obtained for the combination of 50% fresh chondrocyte cultivation medium and 50% conditioned medium derived from 200 mg vital cartilage (Figure S1). The beginning of cultivation in conditioned media was defined as day 0. Medium change was realized every two to three days. The conditioned medium used for the cultivation of the spheroid cultures was resourced from the respective days of cartilage incubation, e.g., conditioned medium recovered after days 8–10 of incubation of VC and HHPTC was used for the cultivation of spheroids at days 8–10. Cultivation was performed at 37 °C, 5% CO_2_ and 21% O_2_. For all experiments, each of the three chondrocyte donors was cultivated with each of the four cartilage donors used for the conditioned medium generation. At day 14 and 35 spheroids were harvested. Each sample was fixed with Formafix^®^ (4% formaldehyde, Grimm Med Logistik GmbH, Torgelow, Germany) for histological analyses (Figure 2).

### 2.7. Histological Analysis

Formalin-fixed samples were washed, dehydrated and embedded in liquid paraffin. The refrigerated samples were cut with a rotary microtome into 5 µm slices. For further staining the slices were deparaffinized in a descending alcohol series. Hematoxylin-eosin staining (HE) was used to get an overview of cell morphology and ECM. The sections were stained with conventional dyes Haemalum by Mayer (Merck, Darmstadt, Germany) and Eosin G (Merck, Darmstadt, Germany) leading to blue appearance of cell cytoplasm and bright red stained extracellular proteins. Additionally, negatively charged structures such as glycosaminglycans of hyaline cartilage were stained blue due to the binding of the positively charged hematoxylin. Alcian-blue staining was used to stain the sulphated glycosaminoglycans of the ECM. The sections were stained with conventional Alcian-blue (Alcian blue 8GS, Chroma, Gujarat, India) in NaCl solution (0.9%) mixed with MgCl_2_ and counterstained with Eosin G (0.1%). Heidenhain’s AZAN trichrome staining was used to stain collagen molecules of the ECM. The sections were stained with conventional dyes azocarmine-G (Sigma-Aldrich, St. Louis, MO, USA), anilin (Sigma-Aldrich) alcohol (90%) and an anilin blue orange G (Merck, Darmstadt, Germany) acetic acid mixture. AZAN staining leads to a blue appearance of synthesized collagen and red cell nuclei.

The quantification of histological staining was performed using the software ImageJ to analyze the area stained with the respective color. A total of 72 images were analyzed by two independent researchers for the Alcian blue staining. For AZAN staining the values varied strongly within the separate groups, especially in the control, an additional analysis of the 72 images was performed by a third independent researcher. Briefly, the area of the slice in the image was roughly selected with the “segmented line selection” tool and the outside cleared. After adjusting brightness and saturation to remove the remaining background, the total area of the slice was measured. Color tresholding was then used to select and measure the blue stained areas for proteoglycans (Alcian blue) or collagen (AZAN) and the red to purple counterstained area of the cell nuclei. Percentage of Alcian blue or AZAN staining was calculated by dividing the blue stained area by the total area of the slice. For statistical analysis the mean of the results from the independent researchers was used to minimize the assessor’s bias.

### 2.8. Immunhistochemical Staining

Performance of immunohistochemical stainings for aggrecan and collagen type II including enzymatic digest was described before [15]. For the specific binding of aggrecan, the monoclonal anti-human Aggrecan G1-IGD-G2 domains primary antibody (1:200, R&D Systems, Minneapolis, MN, USA) was applied and detected by the secondary antibody Alexa Fluor 488 goat anti-mouse IgG (H + L) (1:200, Invitrogen Molecular Probes, Eugene, OR, USA). Cell nuclei were stained with Hoechst (Invitrogen, Carlsbad, CA, USA). For the specific detection of collagen type II, the primary monoclonal mouse anti-chicken collagen type II (IgG) antibody (1:200, Chemicon Int. Inc., Temecula, CA, USA) was used coupled with Alexa Fluor 488 goat anti-mouse IgG (H + L) (1:200, Invitrogen Molecular Probes) as the secondary antibody. Cell nuclei were stained with Hoechst. The quantification was performed using the software ImageJ to analyze the fluorescence signal. For the purpose of background correction the mean fluorescence signal of the antibody control was subtracted from sample signal. Division by area of nuclei is required for sample comparison and determination of protein expression relative to cell quantity.

The following formula was used to calculate the relative fluorescence signal (RFU):

RFU = (Mean fluorescence signal sample − Mean fluorescence signal antibody control)/Area of nuclei.

### 2.9. Gene Expression Analysis by Semiquantitative RT-PCR

Total RNA was isolated from the spheroids with the “peqGOLD Total RNA Kit” (PeqLab/VWR, Erlangen, Germany) for tissue. The procedure was performed according to the manufacturer’s instructions and following final elution, the RNA concentration and quality was determined by measuring the absorbance at 260 nm and 280 nm with a spectrophotometer (Infinite 200 PRO, TECAN, Maennedorf, Switzerland). The isolated RNA was stored at −80 °C before reverse transcription into cDNA. For the reverse transcription, the “High Capacity cDNA Transcription Kit” (Applied Biosystems, Thermo Fisher Scientific, Vilnius, Lithuania) was used. Briefly, the RNA samples (at least 16 ng up to 56 ng RNA) were diluted with diethylpyrocarbonate (DEPC) water to a total volume of 10 µL. A total of 10 µL of master mix was added and PCR was carried out using following RT-PCR protocol: 10 min at 25 °C, 120 min at 37 °C, 15 s at 85 °C in a thermocycler (Analytik Jena, Jena, Germany). Afterwards the samples were diluted with 20 µL DEPC water and stored at −20 °C.

Relative quantification of target genes was performed by semi-quantitative real-time PCR (qTower 2.0, Analytik Jena AG, Jena, Germany) with the innuMIX qPCR MasterMix SyGreen (Analytik Jena AG, Jena, Germany). A master mix was prepared for each gene, containing 0.5 µL of forward and reverse primer (Sigma-Aldrich, Darmstadt, Germany; for sequences refer to Table 2), 2 µL Aqua dest. and 5 µL of SyGreen qPCR MasterMix. A total of 2 µL of template cDNA of each sample was pipetted onto the bottom of a 96-well PCR plate in duplicates and filled up with 8 µL of master mix. RNase-free (DEPC) water served as a negative control. The plate was sealed with adhesive foil and placed in the qTower 2.0. qPCR was performed using the software PCR soft 1.1 under the following conditions: 2 min at 95 °C and 40 cycles of 95 °C for 5 s and 60–65 °C (depending on the primer set) for 25 s each. A cycle threshold (Ct) of 30 was set as the limit of interpretation. The relative expression of each mRNA compared with the housekeeping gene β-actin was calculated by the equation

∆Ct = Ct_target_ − Ct_β-actin_

and depicted as

2^(−∆Ct)^ × 100 (percentage 2^(−∆Ct)^).

### 2.10. Statistical Analysis

If not otherwise stated, data are presented as the arithmetic mean of the samples and the standard deviation as standard error of the mean. A minimum of three independent donors was used to acquire statistical relevant data. The statistical analysis was performed using GraphPad Prism 7.02 (GraphPad Software, San Diego, CA, USA). Normality was tested with Shapiro–Wilk for samples <5000. As values were normally distributed comparisons between two groups were performed with the unpaired Student’s t test or unpaired t test with Welch’s correction depending on variances being significantly different while comparisons between more than two groups were done with One-way ANOVA with Bonferroni’s multiple comparison as post hoc test or for both time points with Repeated Measures Two-way ANOVA with “time” (14 and 35 days) and “treatment” (NC, VC-CM, HHPTC-CM and PC) as variables and Bonferroni’s multiple comparison as post hoc test.

## 3. Results

### 3.1. High Hydrostatic Pressure (HHP) Treatment Leads to Cell Death

**Figure 3:**
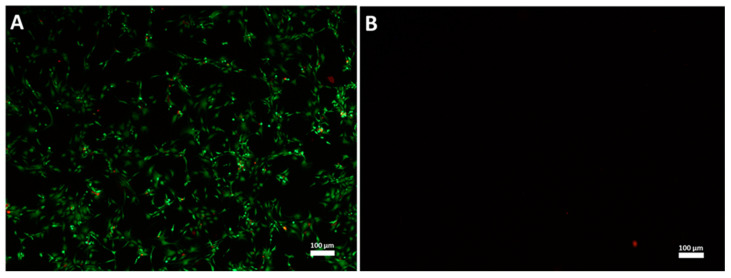
Example of Live/Dead staining of digested cartilage: After the conditioning of medium for 35 days cartilage (vital and pressure treated) was digested and the retrieved cell suspension was cultured for 8 days. Live/Dead staining was performed with cells cultured from digested vital cartilage (VC) (**A**) or high hydrostatic pressure treated cartilage (HHPTC) (**B**). Live cells appear green and dead cells appear red. Magnification: 40×, scale bar: 100 µm.

After HHP treatment of human articular cartilage, vital as well as HPP-treated tissue samples were kept in cell culture medium under appropriate conditions for 35 days to generate conditioned medium, either by active production from living cells or by leaching from the cartilage. To proof the devitalization by HHP, the samples of cartilage were digested with collagenase A and the number of living cells was determined by Trypan blue staining. Only viable cells were counted. After digestion of the cartilage samples an average cell count of 1.4 × 10^6^ viable cells per 100 mg tissue (SD: 1.0 × 10^6^, *n* = 4) were determined at day 35 of incubation in vital cartilage only. Contrary to vital cartilage, HHP-treated cartilage did not contain living cells. The result was confirmed by Live/Dead staining (Figure 3). Thus, both methods verified the successful devitalization of the used cartilage by the high hydrostatic pressure treatment.

### 3.2. Components of Cartilage ECM, Growth Factors and Binding Proteins are Released into Media

**Figure 4:**
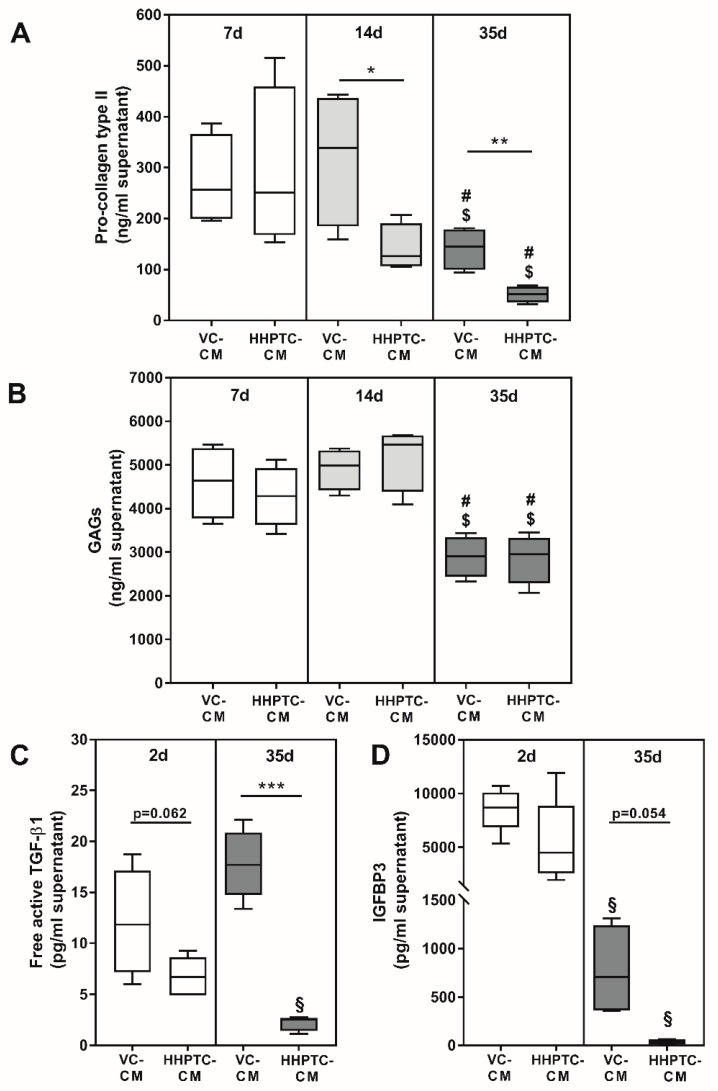
Content of proteins released into conditioned media. Conditioned media (CM) from vital (VC) and high hydrostatic pressure treated cartilage (HHPTC) were analyzed in supernatants generated during days 0 to 2 (2 d), 4 to 7 (7 d), 11 to 14 (14 d) and 32 to 35 (35 d) of cultivation as indicated in the separate graphs. Concentrations of procollagen type II (**A**), free active transforming growth factor (TGF-)β1 (**C**) and insulin-like growth factor-1 binding protein (IGFBP)3 (**D**) were quantified by enzyme-linked immunosorbent assays (ELISA; all *n* ≥ 3). Glycosaminoglycans (GAGs) (**B**) were quantified by Blyscan™ assay after enzymatic digestion with papain (*n* = 4). All concentrations are depicted as weight per volume. Data are presented as boxplots with minimum, 25th percentile, median, 75th percentile and maximum. As data were normally distributed according to Shapiro–Wilk test statistical analysis was performed with unpaired t test or unpaired t test with Welch’s correction depending on variances being significantly different. * *p* < 0.05, ** *p* < 0.01 and *** *p* < 0.001: significance between vital and high hydrostatic pressure treated cartilage. ^§^ *p* < 0.05: significant difference between 2 and 35 days, ^$^ *p* < 0.05: significant difference between 7 and 35 days and ^#^ *p* < 0.05: significant difference between 14 and 35 days for the same treatment.

Prior to analyzing the effect of conditioned media on the re-differentiation of chondrocytes, the constituents of the conditioned media were determined. While samples were collected consecutively every two to three days and then used for the respective days in the spheroid cultures, analyses of constituents were only performed at certain time points, i.e., after 7, 14 and 35 days, due to the extensive number of samples. The concentration of growth factors was only measured in the first (CM 1) and the respective last sample of VC and HHPTC conditioned media (cartilage from four different donors) after 35 days. All determined constituent concentrations are presented in Figure 4 as weight per volume in order to understand which amounts were actually applied in the cultivation of spheroids (0.5 mL conditioned medium plus 0.5 mL fresh chondrocyte cultivation medium without growth factors).

Concentration of the C-terminal pro-peptide of collagen type II in VC conditioned medium was significantly higher compared to HHPTC conditioned medium at time points 14 and 35 days, but not for 7 days (*p* = 0.843, *p* = 0.045 and *p* = 0.007 for 7, 14 and 35 days, respectively, unpaired t test, Figure 4A). While concentrations did not differ between day 7 and 14, there was a decline in the release of C-terminal pro-peptide of collagen type II from cartilage after 14 days (Figure 4A).

Other structural components apart from the collagens such as GAGs also continued to be released from the cartilage into the medium. In general, more GAGs were released into the conditioned medium than the collagen type II, but similar to collagen type II a significant reduction of GAG release was observed after 35 days compared to 7 days (*p* = 0.012 and *p* = 0.020, unpaired t test for VC and HHPTC conditioned medium, respectively, (Figure 4B) as well as to 14 days (*p* = 0.001 and *p* = 0.003, unpaired t test for VC and HHPTC conditioned medium, respectively, Figure 4B). However, the amounts of GAGs did not differ significantly between VC and HHPTC conditioned medium at any of the analyzed time points (Figure 4B).

The concentration of free active transforming growth factor (TGF-)β1 was determined in order to analyze differentiation driving effects of cartilage conditioned media on chondrogenic re-differentiation. We could show that after 35 days VC conditioned medium contained significantly higher concentrations of free TGF-β1 than HHPTC conditioned medium (*p* = 0.0003, unpaired t test with Welch’s correction) (Figure 4C). While there was already a trend at day 2 (*p* = 0.062, unpaired t test) the difference was more pronounced at day 35 due to a significant reduction of free active TGF-β1 from day 2 to day 35 in HHPTC conditioned medium (*p* = 0.001, unpaired t test) but not in VC conditioned medium.

Additionally, concentrations of insulin like growth factor (IGF-)1 and IGF binding protein (IGFBP)3 were determined. IGF-1 protein concentrations were below the detection limit in all conditioned media. Contrary, enhanced protein concentrations of released IGFBP3 in VC compared to HHPTC conditioned medium were determined at day 35, however this difference did not reach significance (*p* = 0.054, unpaired t test with Welch’s correction, Figure 4D). The concentration of IGFBP3 was significantly reduced in both conditioned media over time (*p* = 0.0006 and *p* = 0.035 for VC and HHPTC, unpaired t test with Welch’s correction).

### 3.3. Influence of Conditioned Media in Spheroid Culture

#### 3.3.1. Influence of Conditioned Media on Cell Number in Spheroid Culture

**Figure 5:**
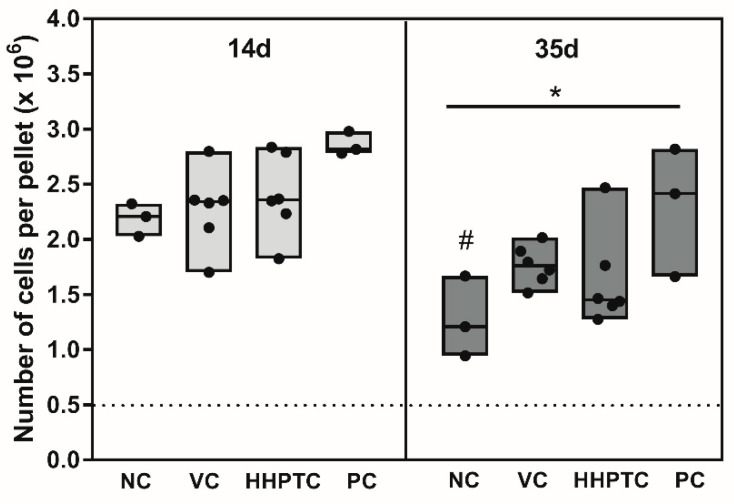
Number of cells per pellet after 14 and 35 days of cultivation: at day 14 and 35 of cultivation number of cells per pellet was determined using CyQuant^®^ Assay. Number of chondrocytes per pellet at day 14 (**left**) and day 35 of cultivation (**right**) differs between used media groups. The following media groups were tested: Negative control (NC), vital cartilage conditioned medium (VC), high hydrostatic pressure treated cartilage conditioned medium (HHPTC) and positive control (PC). The dotted line depicts the initial number of cells. Solid lines within boxes show the median, minimum and maximum, while dots represent the single values. Biological duplicates were used (*n* = 6). Significance was calculated with Repeated Measures Two-way ANOVA with “time” (14 and 35 days) and “treatment” (NC, VC-CM, HHPTC-CM and PC) as variables. Significance: * *p* < 0.05 between treatments and ^#^ *p* < 0.05 comparison between day 14 and 35.

After 14 days in spheroid culture, the number of isolated chondrocytes was approximately 4 to 5 times the number of originally pelleted cells, suggesting successful proliferation of cells in the spheroids. Statistical evaluation with Repeated Measures Two-way ANOVA with “time” (14 and 35 days) and “treatment” (NC, VC-CM, HHPTC-CM and PC) as variables showed a “time” (*p* = 0.0065) as well as a “treatment” (*p* = 0.0325) dependent effect. There were no significant differences regarding the number of cells in NC, PC or conditioned media treated samples at day 14 (Figure 5).

After 35 days of cultivation in the spheroids we observed a decline in cell number compared to 14 days. However, the extent of the decline clearly depended on the cultivation conditions. While conditioned media and growth factors more or less sustained cell survival (Figure 5), there was a significant reduction in cell number in NC (Bonferroni’s multiple comparison as post hoc test: *p* = 0.0387) from 14 to 35 days and cell number on day 35 in NC was also significantly lower than in PC (Bonferroni’s multiple comparison as post hoc test: *p* = 0.0342).

#### 3.3.2. Histological Analyses of Chondrogenic Re-Differentiation in Spheroid Culture

**Figure 6:**
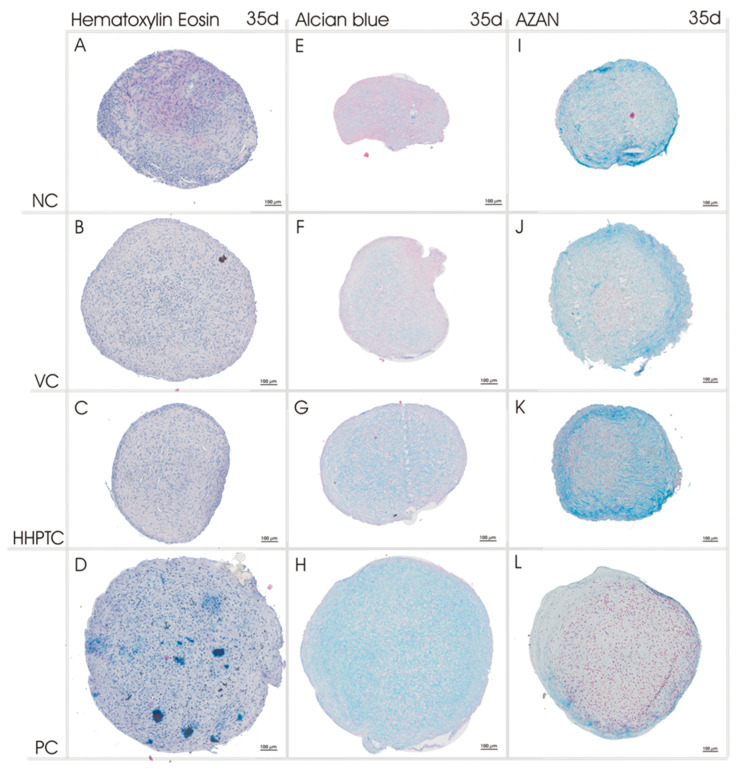
Representative pictures of HE, Alcian blue and Heidenhain’s AZAN trichrome staining of chondrocytic spheroid cultures. Histological staining of chondrocytic spheroid cultures was performed after 35 days of cultivation with different medium groups: Negative control (NC) (**A**,**E**,**I**), vital cartilage conditioned medium (VC) (**B**,**F**,**J**), high hydrostatic pressure treated cartilage conditioned medium (HHPTC) (**C**,**G**,**K**) and positive control (with chondrogenic growth factors, PC) (**D**,**H**,**L**). *n* = 12, scale bar: 100 µm.

For the histological evaluation of re-differentiation spheroids were harvested at day 14 and 35 after cultivation in conditioned media. Exemplary images of representative stains after 35 days of cultivation are shown in Figure 6 (for day 14 and higher resolution see Supplements Figures S2–S4).

HE staining allowed a general survey of the structure of the spheroids. The dark-blue stained nuclei of the cells were evenly distributed throughout in all spheroid cultures (Figure 6A–D).

The high production of proteoglycans in the positive control was confirmed by the Alcian blue staining which showed the strongest overall intensity of blue stain among all spheroids in the positive control (Figure 6H). The other medium groups (negative control and conditioned media) revealed irregular Alcian blue staining of the cone with areas of higher and lower staining intensity. Conditioned media resulted in higher staining intensities in comparison to the negative control. Especially HHPTC conditioned medium led to an increase in intensity until day 35 (Figure 6G).

Spheroids cultured in HHPTC conditioned medium also showed the highest intensity for AZAN stained collagen (Figure 6K). Since AZAN indicated deposited collagen, all spheroid cultures were positive for collagen matrix (Figure 6I–L).

**Figure 7:**
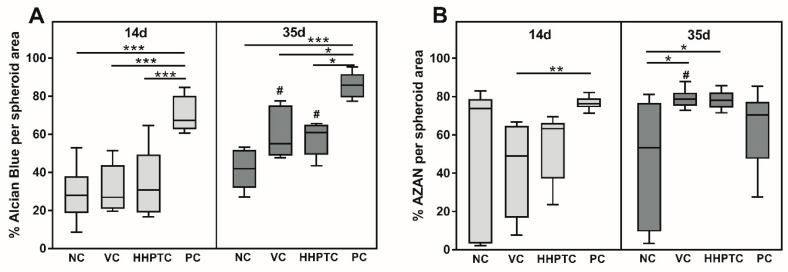
For quantitative analysis of Alcian blue (**A**) and AZAN staining (**B**), the relative blue stained area was determined with ImageJ. For normalization, the measured blue stained area was divided by the total area of the spheroid slice. Data are presented as boxplots with minimum, 25th percentile, median, 75th percentile and maximum. Statistical analysis was performed using Repeated Measures Two-way ANOVA with Bonferroni’s multiple comparison as post hoc test. Significance: * *p* < 0.05, ** *p* < 0.01, *** *p* < 0.001 between treatments and ^#^ *p* < 0.05 comparison between day 14 and 35.

In order to quantify the observed differences, a total of 72 images each were analyzed using the color thresholding of ImageJ to determine the percentage of the blue stained area in relation to the total area of the spheroid slice for Alcian blue as well as AZAN. The results in Figure 7 confirmed the visual observations from Alcian blue staining at day 35: With PC showing the highest percentage of Alcian blue stained area, i.e., proteoglycans, followed by VC and HHPTC conditioned media and with the least stained area in NC. The differences in Alcian blue staining between conditioned media and negative control at 35 days did not reach significance, in particular as only area but not intensity was determined. However, both conditioned media showed a significant accumulation of proteoglycans from day 14 to day 35 (*p* = 0.002 and *p* = 0.017 for VC and HHPTC, respectively, Repeated Measures Two-way ANOVA with Bonferroni’s multiple comparison, Figure 7A) which was not observed for NC. Quantification also verified the highest percentage of AZAN stained areas in spheroids cultivated in conditioned media for 35 days (*p* = 0.014 and *p* = 0.017 for VC and HHPTC vs. NC, Repeated Measures Two-way ANOVA with Bonferroni’s multiple comparison) (Figure 7B).

Especially in the AZAN staining the three-dimensional structure of the spheroids became apparent. The periphery of the spheroids was characterized by the deposition of collagen fibers forming a multilayered cortex or capsule (Figure S3). Furthermore, while this was observed for all spheroids it was most pronounced for the HHPTC conditioned medium (Figure 6K). Furthermore, there was a change in the morphology of the spheroids during cultivation. A decrease in spheroid compactness was observed despite more or less constant or even reduced cell numbers (Figure 5) suggesting that the decline in density was due to the continued deposition of extracellular matrix, either proteoglycans or collagens, between the cells during cultivation.

#### 3.3.3. Influence of Conditioned Media on Gene Expression of Chondrogenic Markers

**Figure 8:**
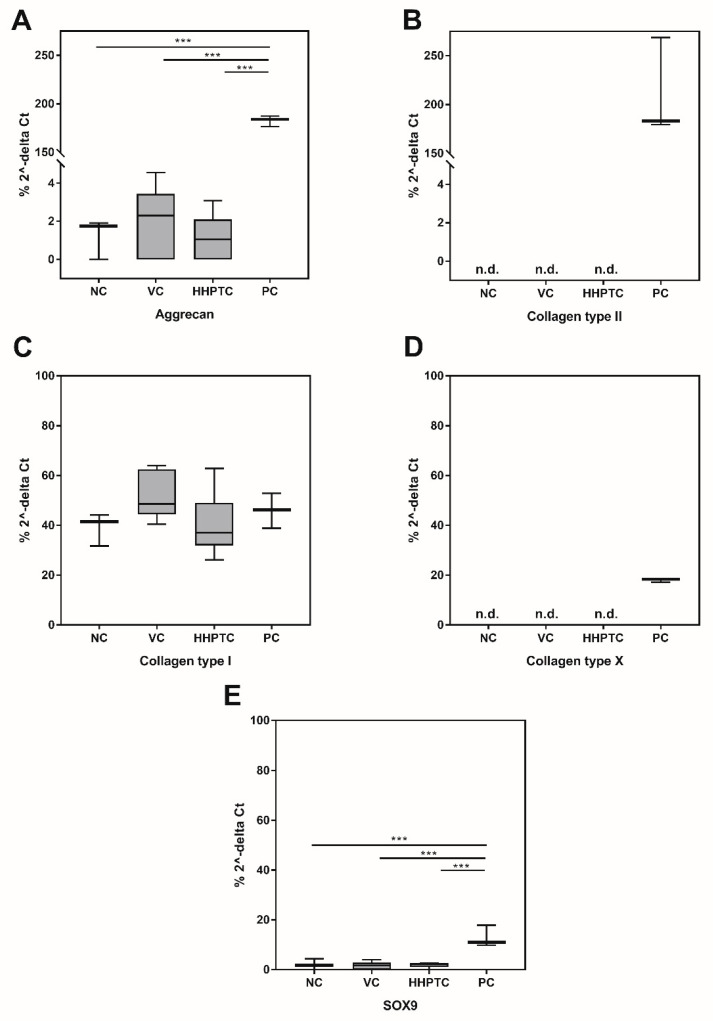
Relative gene expression (percentage of 2^−∆CT^) of relevant chondrogenic genes ((**A**) aggrecan, (**B**) collagen type II, (**C**) collagen type I, (**D**) collagen type X, (**E**) SOX9) in spheroid cultures at day 35. Chondrocyte pellets were cultured in following media: Negative control (NC), vital cartilage conditioned medium (VC), high hydrostatic pressure treated cartilage conditioned medium (HHPTC) and under addition of IGF-1 and TGF-β1 as positive control (PC). Gene expression was determined using semi-quantitative real time PCR. Relative gene expression was calculated based on the internal control (β-actin): ∆Ct = Ct_targe_t − Ct_β-actin_. Statistical evaluation was performed with One-way ANOVA and Bonferroni’s multiple comparison as post hoc test with *n* = 6 for the conditioned media samples and *n* = 3 for controls (NC, PC). Significance: *** *p* < 0.001.

As several of the investigated genes were not expressed on mRNA level in the negative control (NC) it was only possible to relate values of the specific genes to the internal control (β-actin) and values are therefore depicted as percentage of 2^−∆CT^ (Figure 8A–E).

The evaluation of the gene expression data of chondrocytes from the spheroids at day 35 of treatment showed that *COL1A1* (Figure 8C) was expressed at a constant level in the spheroids and that this level was independent from the treatment, i.e., gene expression did not change compared to untreated samples (NC) when the spheroids were treated with conditioned media (VC or HHPTC) or the growth factor mix (PC).

The gene expression of the transcription factor SOX9 (Figure 8E), which plays an essential role in chondrocyte differentiation, and of the chondrocyte-specific ECM component aggrecan (Figure 8A) was detected in all treatment groups as well as in the NC. However, expression was significantly higher after continuous treatment with growth factors IGF-1 and TGF-β1, while there was no difference between NC and spheroids cultivated in conditioned media (one-way ANOVA with Bonferroni’s multiple comparison as post hoc test: *p* = 0.0002, *p* < 0.0001 and *p* < 0.0001 for NC, VC and HHPTC each versus PC for SOX9 as well as *p* < 0.0001, *p* < 0.0001 and *p* < 0.0001 for NC, VC and HHPTC each versus PC for aggrecan, Figure 8A,E, respectively). Gene expression of *COL2A1* was only detectable after stimulation with growth factors IGF-1 and TGF-β1 (Figure 8B). This indicates that only the treatment with the growth factors significantly induced gene expression of chondrogenic markers while the cultivation with conditioned media showed no benefit above and beyond 3D cultivation alone when analyzing gene expression data.

However, it is important to note that the stimulation with the growth factors (PC) also induced the gene expression of the hypertrophy marker *COL10A1* (Figure 8D).

#### 3.3.4. Immunohistochemical Analyses of Collagen Type II and Aggrecan

**Figure 9:**
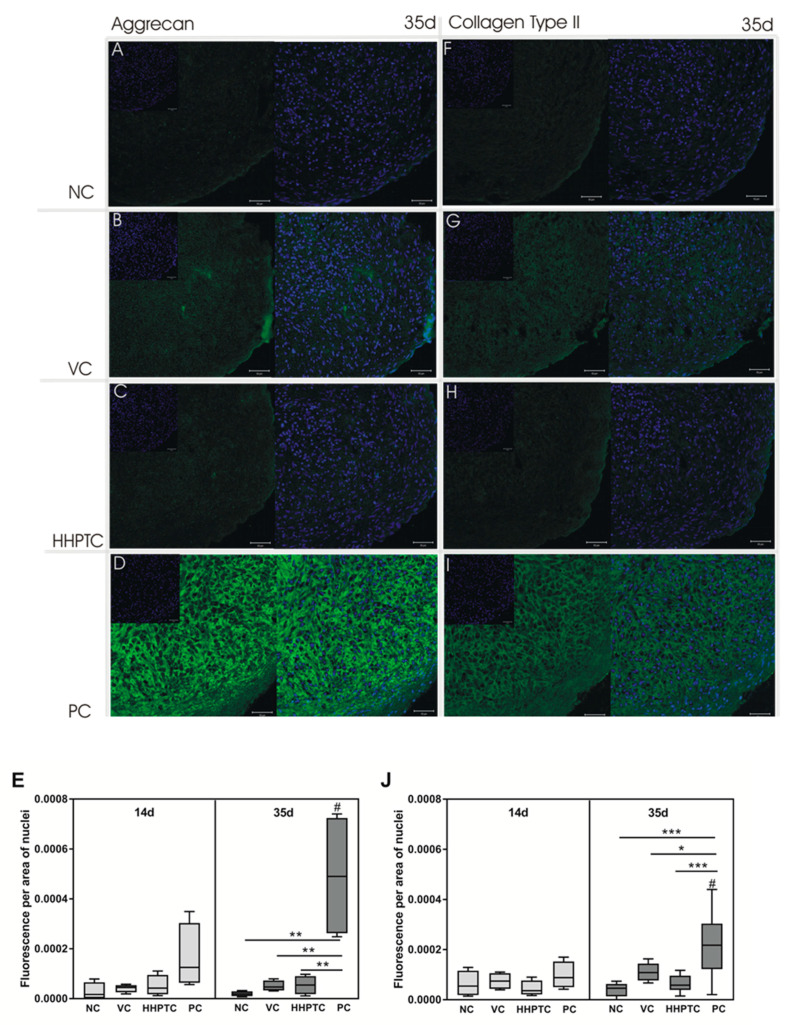
Aggrecan and collagen type II protein expression. Immunohistochemical staining of chondrocytic spheroid cultures at days 14 and 35 was performed using anti-human aggrecan and anti-chicken collagen type II antibody (green fluorescence). Nuclei were stained with Hoechst (blue fluorescence). Chondrocytic spheroids were cultivated in following media groups: Negative control (NC) (**A**,**F**), vital cartilage conditioned medium (VC) (**B**,**G**), high hydrostatic pressure treated cartilage conditioned medium (HHPTC) (**C**,**H**) and positive control (PC) (**D**,**I**). Exemplary images from day 35 depict aggrecan (**A**–**D**) and collagen type II (**F**–**I**) staining. Magnification: 200×, scale bar: 50 µm. For semi-quantitative analysis (**E**,**J**), the relative fluorescence signal was determined with ImageJ. After background correction, mean fluorescence signal was related to area of nuclei (*n* = 8). Data are presented as boxplots with minimum, 25th percentile, median, 75th percentile and maximum. Statistical analysis was performed using Repeated Measures Two-way ANOVA with Bonferroni’s multiple comparison as post hoc test. Significance: * *p* < 0.05, ** *p* < 0.01, *** *p* < 0.001 between treatments and ^#^ *p* < 0.05 comparison between day 14 and 35.

Immunohistochemical analyses of the prominent ECM components aggrecan and collagen type II were performed to investigate the protein expression and distribution within spheroid cultures. Exemplary images from day 35 are depicted in Figure 9A–D (aggrecan) and Figure 9H,I (collagen type II). It has to be taken into account that all images were taken with the same exposure time, thus making it difficult to visualize aggrecan and collagen type II in the images of samples with lower expression such as spheroids after cultivation in conditioned medium of vital cartilage (VC) or of high hydrostatic pressure treated cartilage (HHPTC). Aggrecan as well collagen type II protein were synthesized in all spheroid culture groups. Despite gene expression for *COL2A1* being below the detection limit, the immunohistochemical staining clearly illustrated the distribution and structure of synthesized collagen type II network (Figure 9F–I). The semi-quantitative analysis of the fluorescence signal allowed a comparison of protein production after cultivation in conditioned medium of vital cartilage (VC), of high hydrostatic pressure treated cartilage (HHPTC) and in controls (negative and positive). Repeated Measures Two-way ANOVA of the semi-quantitative analyses with “time” (14 and 35 days) and “treatment” (NC, VC-CM, HHPTC-CM and PC) as variables revealed a significant “treatment” effect for aggrecan as well as collagen II protein expression (*p* < 0.0001 and *p* = 0.0009 for aggrecan and collagen II, respectively). An accumulation over time of aggrecan and less so of collagen type II was observed exclusively in the positive control between 14 and 35 days (*p* = 0.0132 and *p* = 0.0271 for aggrecan and collagen II, respectively). The positive control at day 35 (Figure 9D) also showed the highest fluorescence signal with the typical structural arrangement of aggrecan produced by cultivated chondrocytes compared to negative control, cultivation in VC conditioned medium or HHPTC conditioned medium (Bonferroni’s multiple comparison as post hoc test: *p* = 0.0018, *p* = 0.0028 and *p* = 0.0030 for NC, VC and HHPTC each versus PC, respectively, Figure 9E). A similar result was also observed for collagen type II protein in the spheroids at day 35 (Bonferroni’s multiple comparison as post hoc test: *p* < 0.0001, *p* = 0.0004 and *p* = 0.0143 for NC, VC and HHPTC each versus PC, respectively, Figure 9J). There were no significant differences between NC, VC and HHPTC at day 35, neither for aggrecan nor for collagen type II.

#### 3.3.5. Influence of Conditioned Media on TGF-β1 Release from Chondrocytic Spheroid Cultures

In order to determine further effects of conditioned media on the re-differentiation of chondrocytes, chondrocytic spheroid cultures were cultivated in the different media and the release of active TGF-β1 at day 35 of spheroid culture was measured. The determined values represent the concentration of free active TGF-β1 in the supernatants of the spheroid cultures minus the concentration of free active TGF-β1 found in the conditioned media before they were added to the spheroids. The thus determined values were 1.9 ± 2.5 pg/mL (total including concentration in conditioned medium was 14.9 ± 4.2 pg/mL) after cultivation in VC conditioned medium, 11.4 ± 4.3 pg/mL in HHPTC conditioned medium (total including concentration in conditioned medium was 13.7 ± 4.2 pg/mL) and 12.7 pg/mL in negative control (culture medium only, total including concentration in culture medium was 12.8 pg/mL, pooled samples). The difference between spheroid cultures cultivated in VC and HHPTC conditioned medium was statistically significant (*p* = 0.001, unpaired t test).

## 4. Discussion

Since the prevalence of cartilage defects and the risk of disease progression towards osteoarthritis are still increasing, sufficient treatment strategies for articular cartilage defects are required [35]. Several strategies including the application of cells during autologous chondrocyte implantation (ACI) have been applied in orthopedic surgery. However, the major disadvantage of ACI is the de-differentiation and phenotype change of chondrocytes [36] originating from the cell expansion during ex vivo cultivation [3]. Therefore, finding ideal cultivation conditions for the induction of cell re-differentiation has already been the goal of several studies including those of our working group [15,16,17]. In the present study, we hypothesized that conditioned media from vital (VC) and high hydrostatic pressure treated cartilage (HHPTC) may have chondrogenic effects in chondrocytic 3D spheroid cultures.

### 4.1. Analyses of VC and HHPTC Conditioned Media

In this study conditioned media were generated by cultivation of 200 mg cartilage (VC or HHPTC) per mL medium whereas the control consisted of media without the addition of cartilage tissue. Apparently, the inactivation of chondrocytes during high hydrostatic pressure treatment did not influence the release of sulphated GAGs by cartilage tissue compared to vital cartilage. Vice versa, these results indicated that cells within the vital cartilage tissue did not synthesize new GAGs and the number of released GAGs declined over time. The main reasons for this finding could be the small number of cells within vital cartilage, the artificial ex vivo environment in cell culture and a changed cell metabolism within pathological processes in osteoarthritic cartilage. While the release of c-terminal pro-peptides of collagen II also declined between day 14 and 35 there was a significantly higher release of procollagen type II for VC on day 14 as well as day 35 compared to HHPTC. This confirmed the presence of metabolic active cells with synthesis capacities solely in vital cartilage; however, these cells newly synthesized only the collagen matrix but no GAGs. Since we were also able to detect GAGs and procollagen molecules in HHPTC, we hypothesized that these matrix components were partly released by the ECM itself. The high pressure treatment may cause the cleavage of collagen, thus releasing the C-terminal pro-peptide into the extracellular space where it is captured in the ECM [37] and only slowly released in the supernatant due to the high density of the ECM. Furthermore, the high hydrostatic pressure treatment led to inactivation and cell death which was accompanied by a release of intracellular components. Therefore, intracellular collagen may also be released by HHPTC into the medium, resulting in a detection of collagen type II. Despite a reduction in the protein concentration of procollagen type II in the conditioned medium harvested at day 35 compared to the conditioned medium harvested at day 14 of incubation, the concentration at day 35 was still higher than in the unconditioned medium, therefore supporting the assumption of a long-term ECM release from the HHPTC.

Our results demonstrated a typical phenotype for articular cartilage regardless of cartilage tissue not being cultured under physiological conditions, such as reduced oxygen levels or biomechanical stimulation, which are crucial for maintenance of homeostasis [38].

Apart from matrix components, growth factors, which are released to regulate homeostasis in vivo [39], may also be deposited in the ECM. Liu et al. showed low levels of IGF-1 and TGF-β1, 2 and 3 as well as high levels of IGF-2, FGF-4 and IGFBP4 and 6 in human costal chondrocyte-scaffold derived conditioned media [30]. They assumed that these and further factors may induce chondrogenic differentiation of mesenchymal stem cells derived from bone marrow [30]. In our study, we analyzed the release of free active TGF-β1, IGF-1 and IGFBP3. Free active TGF-β1 varied between the conditioned media groups and was found in significantly higher amounts in VC. The failure to detect IGF-1 may be attributed to the use of osteoarthritic cartilage. As reported by Martin et al. [40] an enhanced proportion of IGF-binding protein 3 (IGFBP3) and fibronectin was found in OA cartilage, both of which control the release of IGF-1 [39]. Indeed, we measured increased amounts of IGFBP3 in our conditioned media which could in term scavenge free IGF-1 protein and making it thus undetectable.

Moreover, other growth factors or chemokines/cytokines in the conditioned media are expected to vary compared to healthy cartilage due to mediators released in OA such as interleukin (IL-)1 and 6, tumor necrosis factor (TNF-)α or matrix-metalloproteases (MMP) [41]. When osteoarthritic cartilage is used further analyses are necessary to examine both pro-chondrogenic and inflammatory mediators in the cartilage conditioned media.

### 4.2. Induction of Hyaline Matrix Deposition by Conditioned Media

In all spheroids, independent from the cultivation conditions, the deposition of proteoglycans and to a lesser degree of collagen type II was observed. Whereas supplementation with growth factors resulted in a massive induction of extracellular matrix production, the effects in 3D cultivation of human chondrocytes with and without simultaneous stimulation with conditioned media were less pronounced. Semi-quantitative analyses of protein deposition of aggrecan and collagen type II and gene expression of chondrogenic genes did not differ between chondrocytes cultivated in VC or HHPTC conditioned media nor did these analyses show a significant benefit of incubation with conditioned media compared to negative control. Our lack to observe significant effects on gene and protein expression with the conditioned media beyond the negative control might be partly due to the fact that already the cultivation of chondrocytes in pellet cultures was shown to effectively induce re-differentiation [42] and to maintain the matrix composition of hyaline cartilage [43]. Similar to our results with the conditioned media, Stewart et al. showed that while the addition of rhBMP-2 significantly increased the production of collagen type II and aggrecan in monolayer cultures of equine articular chondrocytes, in the pellet cultures there was little response, mainly because pellet cultures already favored the chondrocytic phenotype [44]. It is also possible that the high density of cells and the extracellular matrix in the spheroids hinders the uptake of extrinsic growth factors as cyclic compression, which aids diffusion in such a compact environment [45] was not exerted in our culture system. Still, the cultivation in conditioned media seems to have favorable effects as both media were able to suppress the decline in cell number observed in the negative control. Furthermore, the histological stainings where, with the exception of the positive control, the highest intensities of Alcian Blue and AZAN staining were observed in conditioned media cultured spheroids hinted at additional benefits of the conditioned media compared to negative control. Even more interesting was the observation that the AZAN staining of the spheroids, especially after cultivation with the conditioned media, unveiled a three-dimensional zonal organization of collagen fibrils that was similar to the one described for healthy articular cartilage with superficial tangential zone, middle zone and deep zone [38]. This zonal organization was completely absent in the growth factor stimulated spheroids.

As major contributors, growth factors were proven to initiate re-differentiation since the cultivation of chondrocytes in 3D spheroid cultures with IGF-1 and TGF-β1 resulted in an elevated expression of typical ECM components [16]. Thus, as a positive control we stimulated spheroid cultures with TGF-β1 and IGF-1 as previously described [16,18,19] to compare the impact of conditioned media and of sole growth factor supplementation on chondrogenic re-differentiation. In contrast to the spheroids cultivated in conditioned media the positive control showed a tendency for a change towards a hypertrophic phenotype in the histological staining and the induction of the gene expression of hypertrophy marker collagen type X. This effect was also observed in other studies, in which the use of TGF-β1 increased the expression of hypertrophy related genes [46]. On the contrary Witt et al. [19] showed that supplementation with TGF-β1 in chondrocytic spheroid cultures did not result in the induction of hypertrophy marker expression. This could indicate that the TGF-β1 effects are dose-dependent. Furthermore, the different observations may be caused by the use of non-degenerative cartilage [19] compared to the osteoarthritic cartilage in our study. Cultivation of chondrocytes in conditioned media did not result in unwanted morphological changes, probably due to the relatively low concentrations of TGF-β1 in our conditioned media. The analyses of the supernatants of the respective spheroid cultures showed varying concentrations of active TGF-β1. Interestingly, chondrocytic spheroid cultures stimulated with HHPTC conditioned media released higher amounts of TGF-β1 compared to cultures stimulated with VC conditioned media. This result could indicate a negative feed-back loop of cellular growth factor production: The more TGF-β1 is already present in the conditioned media the less will be produced by cells and vice versa. In this context it is interesting to note that the total concentration of active TGF-β1 in the supernatants of spheroids was with approximately 13–15 pg/mL almost identical for the culture in negative control, VC conditioned medium and HHPTC conditioned medium, suggesting that the TGF-β1 concentration is adjusted by the chondrocytes at that level. This is in accordance with the in vivo data by Albro et al. [47] who determined a secretion rate of 50–80 pg/million cells per day for latent TGF-β1 in articular cartilage. When considering that less than 10% of the secreted TGF-β1 is in the active form [25] and based on the cell number of around two million in our spheroids we are in the range of 10–16 pg of secreted active TGF-β1, i.e., similar to the range in our spheroid cultures. Albro et al. also showed that TGF-β1 level in explants remained constant over time, thus indicating that under physiological conditions TGF-β1 production is tightly regulated and reaches an equilibrium [47]. The thus adjusted TGF-β1 concentration might be ideal to maintain the composition of a healthy, hyaline matrix. Therefore, the almost identical, total TGF-β1 concentration in culture supernatants of spheroids cultivated in negative control, VC conditioned medium and HHPTC conditioned medium might be another reason for the similar gene and protein expression of aggrecan and collagen type II. In contrast, the amount of TGF-β1 in spheroid supernatants after synthetic growth factor stimulation was approximately 400 times higher than in cartilage conditioned media indicating a massive overstimulation of cells. Referring to results from Jonitz et al. [16] and Indrawattana et al. [48], who analyzed the effects of IGF-1 alone, IGF-1 is assumed to increase proliferation and to enhance effects of TGF-β when combined. IGF-1 may also support terminal differentiation effects of TGF-β by hypertrophic differentiation and mineralization [46]. This might be a reason for phenotypical changes in the positive control shown in our histological staining. Nevertheless, using conditioned medium instead of synthesized growth factor supplemented medium might enable the generation of several biochemical and physical cues at a more physiological dose [29] and the mixture of conditioned medium with fresh cultivation medium decreases the concentration of catabolic factors and consequently the probability of inhibitory side effects. However, as the concentration of TGF-β1 is similar in the supernatants amongst negative control and in VC or HHPTC conditioned media cultivated spheroids, we assume that not TGF-β1 but the additional matrix components in the conditioned media might account for the slight improvement in spheroids cultured in conditioned media.

One potential factor involved in the process of chondrogenesis is the collagen in the conditioned media as collagen induces differentiation and re-differentiation when chondrocytes are cultured on collagen matrices [49]. The interaction of chondrocytes with matrix molecules is realized by their integrin cell adhesion receptors, which bind ECM proteins like fibronectin and collagen type II, thereby initiating signal transduction. Not only the intact matrix but also fragments have been shown to provide this interaction [50]. This puts emphasis on our assumption that the released collagens exhibit a chondrogenesis promoting effect. The slightly enhanced chondrogenic re-differentiation of cells cultivated with VC conditioned medium in our study is likely to be related to the increased levels of collagen found in this particular conditioned medium. Moreover, our data are in accordance with results from Gavenis et al. [49], who showed that a combination of high collagen type II and low collagen type I levels in a collagen gel system led to enhanced re-differentiation of chondrocytes. Promoting effects of single collagens were found by Rutgers et al. [51], who reported an elevated production of GAG by human chondrocytes cultured on collagen type I as well as type II coated cell culture inserts. Furthermore, Levingstone et al. [52] showed a positive effect of a collagen scaffold composed of a combination of collagen type I and II and hyaluronic acid on proliferation and chondrogenic differentiation of chondrocytes [52].

Additionally, the released GAGs in the conditioned media may also exert chondroinductive effects. Especially, aggrecan was shown to be essential for the formation of cartilaginous tissue and chondrocyte organization during embryonic development [53]. It induced chondrogenic differentiation in skin cells [54,55] and, similar to chondroitin sulfate, it improved viability and GAG production of chondrocytes in interpenetrating network hydrogels [56]. This chondroinductiveness of some GAGs might explain why HHPTC conditioned medium was also effective in promoting re-differentiation in spheroids as GAG content did not differ between VC conditioned medium and HHPTC conditioned medium.

We are aware of several limitations in our study. Due to the large number of samples a cumulative analysis of the main constituents of the conditioned media for all sampling time points was not feasible. We only gained insight into the composition of the conditioned media at certain time points, but we cannot really determine whether the chosen time points were representative. What we are able to conclude from the few time points is that for the conditioned media we had differing concentrations of components throughout the 35 days of cultivation of the spheroids whereas for the controls, especially the positive control, a constant composition was maintained throughout the whole cultivation time. Furthermore, due to the use of human material inter-individual variation was large and the additional effects of cultivation with conditioned media seldom reached significance when compared to the re-differentiation already achieved by high density 3D cultivation (NC). While the quantification of the histological images showed the highest amounts for AZAN staining of collagen in the spheroids cultivated in conditioned media, we cannot rule out that this increase is due to the production of other collagen types, e.g., collagen type I, as AZAN staining is unspecific and we did not test for further specific collagen types. It would also have been interesting to support the observations of the organized zonal structure of the extracellular matrix in CM-cultivated spheroids with biomechanical data regarding the strength of the spheroids.

Despite these shortcomings, minor benefits on chondrocyte re-differentiation, that were similar between the VC and HHPTC conditioned medium, were observed for cultivation with conditioned media. In general, the generation of conditioned medium with non-degenerative hyaline cartilage is preferable to reduce the enrichment of inhibitory or inflammatory mediators in future studies. Thinking ahead, HHP treatment provides the opportunity for the inactivation and destruction of possible pathogens and antigens during high hydrostatic pressure processing [33], thus allowing the consideration of clinical translation for the generation of conditioned media with devitalized cartilage. The effects of HHPTC conditioned medium could probably be enhanced by concentrating secreted molecules by processes such as lyophilization. In preliminary tests, we were able to increase the protein content by a factor of three through freeze-drying. The higher concentration of chondrogenic factors might have a beneficial impact on re-differentiation. Furthermore, it may be more suitable to pool the conditioned media as we clearly showed that the concentration of active components, which were released into the conditioned media, decreased over time. Pooling the conditioned media and then using aliquots of the pooled sample for cultivation of spheroids would guarantee constant levels of active components. However, this methodological approach including the benefits of freeze-drying has to be verified in further experiments. Apart from flushing the chondrogenic factors out of the HHP-treated cartilage to generate conditioned media, an alternative approach would be to use the HHP-treated cartilage as allografts, as the long-term release of potential chondrogenic factors in our study confirms that there are enough of those factors present in the HHP-treated cartilage, thus allowing the inserted allograft to be colonized in vivo and to grow into the surrounding tissue.

## 5. Conclusions

Long-term incubation of hyaline cartilage in defined cell culture medium led to the release of molecules, such as GAGs, procollagen type II and TGF-β1, resulting in the conditioning of culture medium. Differences were shown in the composition of conditioned media depending on the cartilage used. Further experiments have to be performed to determine additional pro-chondrogenic or catabolic factors. Effects of cartilage conditioned media were tested on chondrogenic re-differentiation in 3D spheroid cultures. Based on histological and immunohistochemical staining the induction of expression of cartilage markers, such as aggrecan and collagen type II, were shown. While the accumulation of the extracellular matrix was already induced by cultivation in high density (spheroid) culture alone, minor additional effects on hyaline-like matrix deposition by the conditioned media may be partly due to released molecules like GAG and procollagens, but also due to cell originated factors in the chondrocytes. However, to finally determine whether the matrix or cellular components released from the cartilage cause the re-differentiation promoting effects, further investigations are required including biomechanical testing of the spheroids.

## Figures and Tables

**Table 1:** Composition of tested media.

Components	Chondrocyte Cultivation Medium/NC	PC	VC-CM	HHPTC-CM
VC-CM (in µL)	-	-	500	-
HHPTC-CM (in µL)	-	-	-	500
DMEM (in µL), supplemented with 1% pen/strep, 1% fungicide	1000	1000	500	500
ITS™	1:100	1:100	1:100	1:100
Ascorbic acid	50 µg/mL	50 µg/mL	50 µg/mL	50 µg/mL
Dexamethasone	100 nM	100 nM	100 nM	100 nM
IGF-1	-	50 ng/mL	-	-
TGF-β1	-	50 ng/mL	-	-

Notes: ITS™ (Insulin-transferrin-sodium selenite media supplement), NC (negative control) PC (positive control), VC-CM (Vital cartilage conditioned medium), HHPTC-CM (High hydrostatic pressure treated cartilage conditioned medium).

**Table 2:** Primer sequences for qPCR.

Transcript	Forward Primer (5′-3′)	Reverse Primer (5′-3′)
β-Actin	CTTCCTGGGCATGGAGTC	AGCACTGTGTTGGCGTACAG
Aggrecan	ACAAGGTCTCACTGCCCAAC	AATGGAACACGATGCCTTTC
Collagen 1 (Col1A1)	ACGAAGACATCCCACCAATC	AGATCACGTCATCGCACAAC
Collagen 2 (Col2A1)	AATGGTGGCTTCCATTCAG	GTGATGTTCTGGGAGCCTTC
Collagen 10 (Col10A1)	GAACTCCCAGCACGCAGAATC	AGTGGGCCTTTTATGCCTGT
SRY-box 9 (Sox9)	AGTACCCGCACCTGCACAAC	CGCTTCTCGCTCTCGTTCAG

## Supplementary Materials

The following are available online at https://www.mdpi.com/2077-0383/9/9/2798/s1, Figure S1: Influence of different concentrations of conditioned medium on the proliferation of chondrocytes in monolayer cultures. Different amounts of vital cartilage (VC, 100 mg or 200 mg per mL medium) were incubated to generate the conditioned medium. Isolated chondrocytes were cultured in a monolayer with conditioned medium, which was either mixed with fresh chondrocyte cultivation medium or left unmixed. The following dilution ratios were used: 100, 70, 50, 30% of CM. (A) Proliferation of cells, which were treated with the different concentrations, was tested using WST-1 (*n* = 3). Data are shown as percentage of negative control (NC = chondrocytes cultivated solely in fresh chondrocyte cultivation medium = 100% proliferation [dotted line]). Significance: * *p* < 0.05 between different concentrations and ^#^ *p* < 0.05 compared to NC. Representative images of live/dead staining are presented in (B): 50% CM from 100 mg cartilage /mL medium with 50% fresh medium, (C): 100% CM from 100 mg cartilage/mL medium, (D): 50% CM from 200 mg cartilage/mL medium with 50% fresh medium, (E): 100% CM from 200 mg cartilage/mL medium. Live/Dead staining was performed with cultivated chondrocytes. Living cells appear green, dead cells appear red. Magnification: 40x, scale bar: 100 µm. Figure S2: Representative pictures of HE staining of chondrocytic spheroid cultures. Figure S3: Representative pictures of Alcian blue staining of chondrocytic spheroid cultures. Figure S4: Representative pictures of Heidenhain’s AZAN trichrome staining of chondrocytic spheroid cultures.
