# Supplementary material for: Influence of Conditioned Media on the Re-Differentiation Capacity of Human Chondrocytes in 3D Spheroid Cultures
Source: J Clin Med. 2020 Aug 30;9(9):2798. doi: 10.3390/jcm9092798 (PMC7564315; doi:10.3390/jcm9092798)
Supplement: Supplementary file 1 [file jcm-09-02798-s001.zip › Supplementary figures/Supplementary figures.docx]

**Supplementary figures S1, S2, S3 and S4:**

**A**


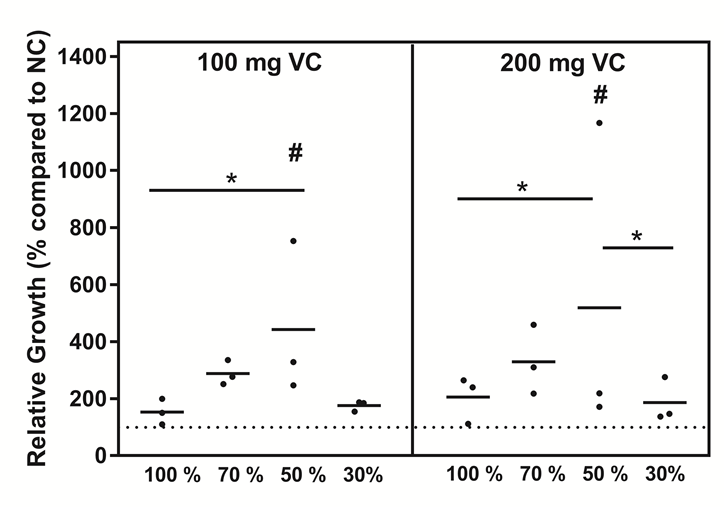


| **B**  **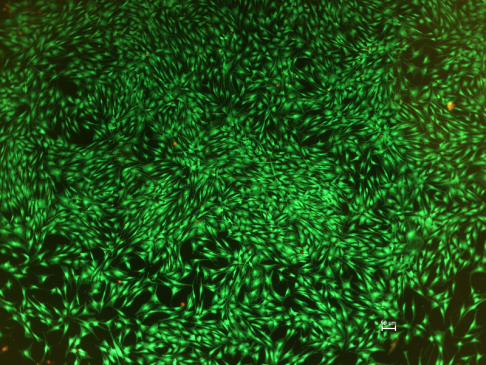** | **C**  **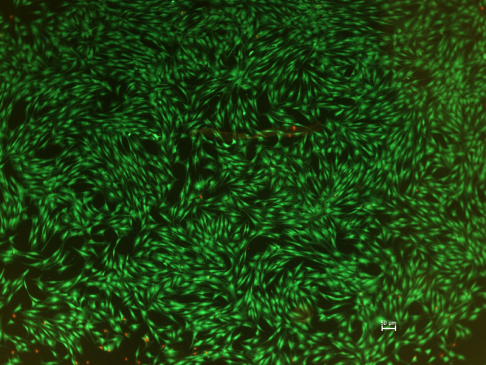** |
| --- | --- |
| **D**  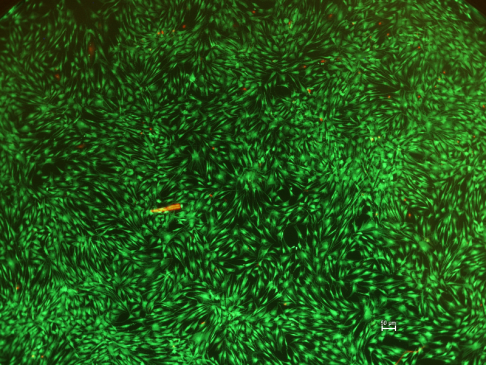 | **E**  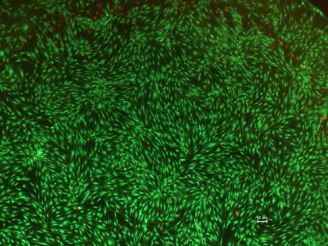 |

Figure S1: Influence of different concentrations of conditioned medium on the proliferation of chondrocytes in monolayer cultures. Different amounts of vital cartilage (VC, 100 mg or 200 mg per ml medium) were incubated to generate the conditioned medium. Isolated chondrocytes were cultured in a monolayer with conditioned medium, which was either mixed with fresh chondrocyte cultivation medium or left unmixed. The following dilution ratios were used: 100, 70, 50, 30 % of CM. (A) Proliferation of cells, which were treated with the different concentrations, was tested using WST-1 (n = 3). Data are shown as percentage of negative control (NC = chondrocytes cultivated solely in fresh chondrocyte cultivation medium = 100 % proliferation [dotted line]). Significance: * p < 0.05 between different concentrations and # p < 0.05 compared to NC. Representative images of live/dead staining are presented in (B): 50% CM from 100 mg cartilage /ml medium with 50 % fresh medium, (C): 100 % CM from 100 mg cartilage /ml medium, (D): 50 % CM from 200 mg cartilage /ml medium with 50 % fresh medium, (E): 100 % CM from 200 mg cartilage / ml medium. Live/Dead staining was performed with cultivated chondrocytes. Living cells appear green, dead cells appear red. Magnification: 40x, scale bar: 100 µm

**
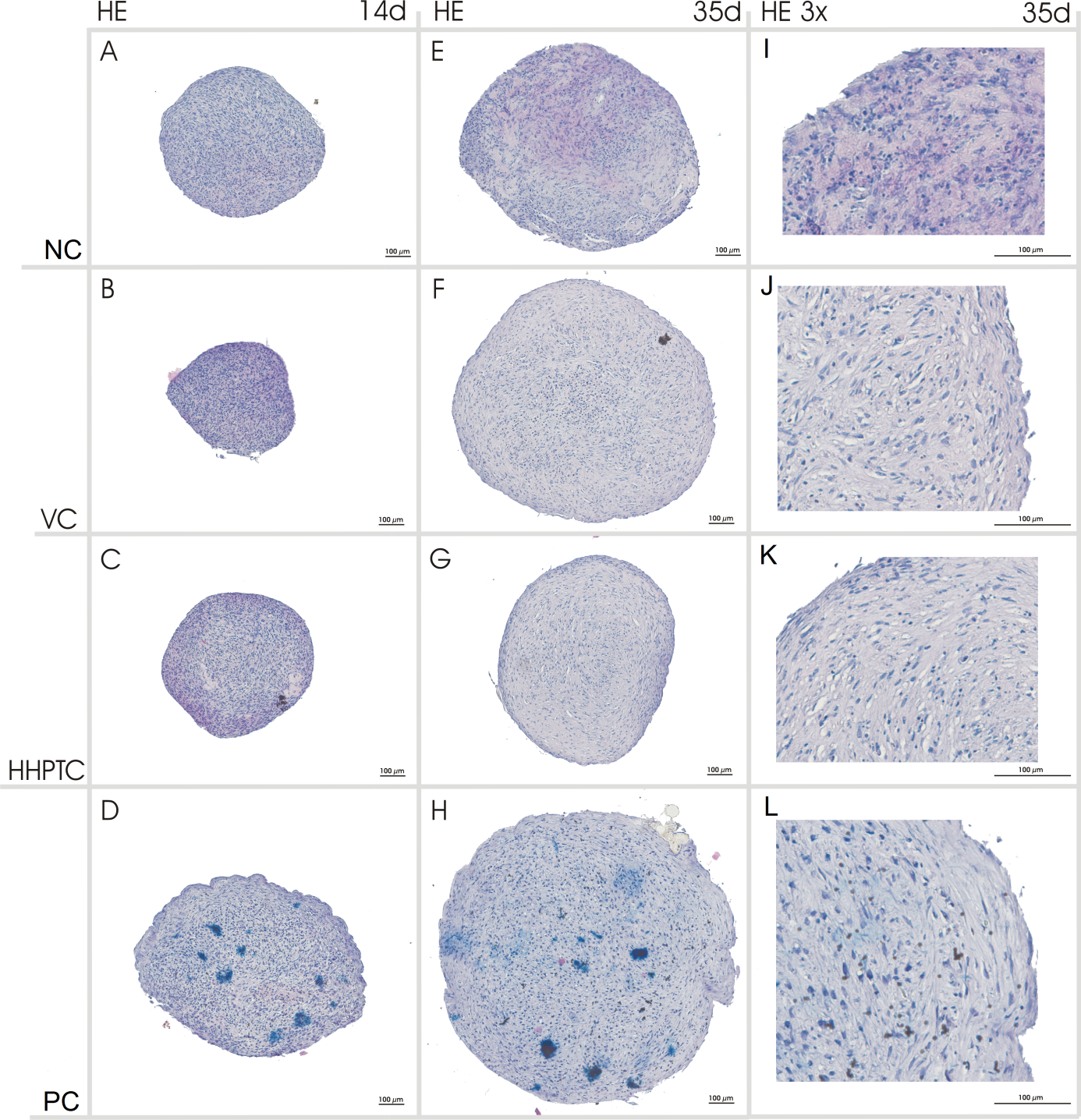
**

**Figure S2: Representative pictures of HE staining of chondrocytic spheroid cultures.** Histological staining of chondrocytic spheroid cultures was performed after 14 days (A-D) and 35 days (E-L) of cultivation with different medium groups: negative control (NC) (A,E,I), vital cartilage conditioned medium (VC) (B,F,J), high hydrostatic pressure treated cartilage conditioned medium (HHPTC) (C,G,K) and positive control (with chondrogenic growth factors, PC) (D,H,L). Three times magnified sections from stains E-H are depicted in pictures I-L. n=12, scale bar: 100 µm.


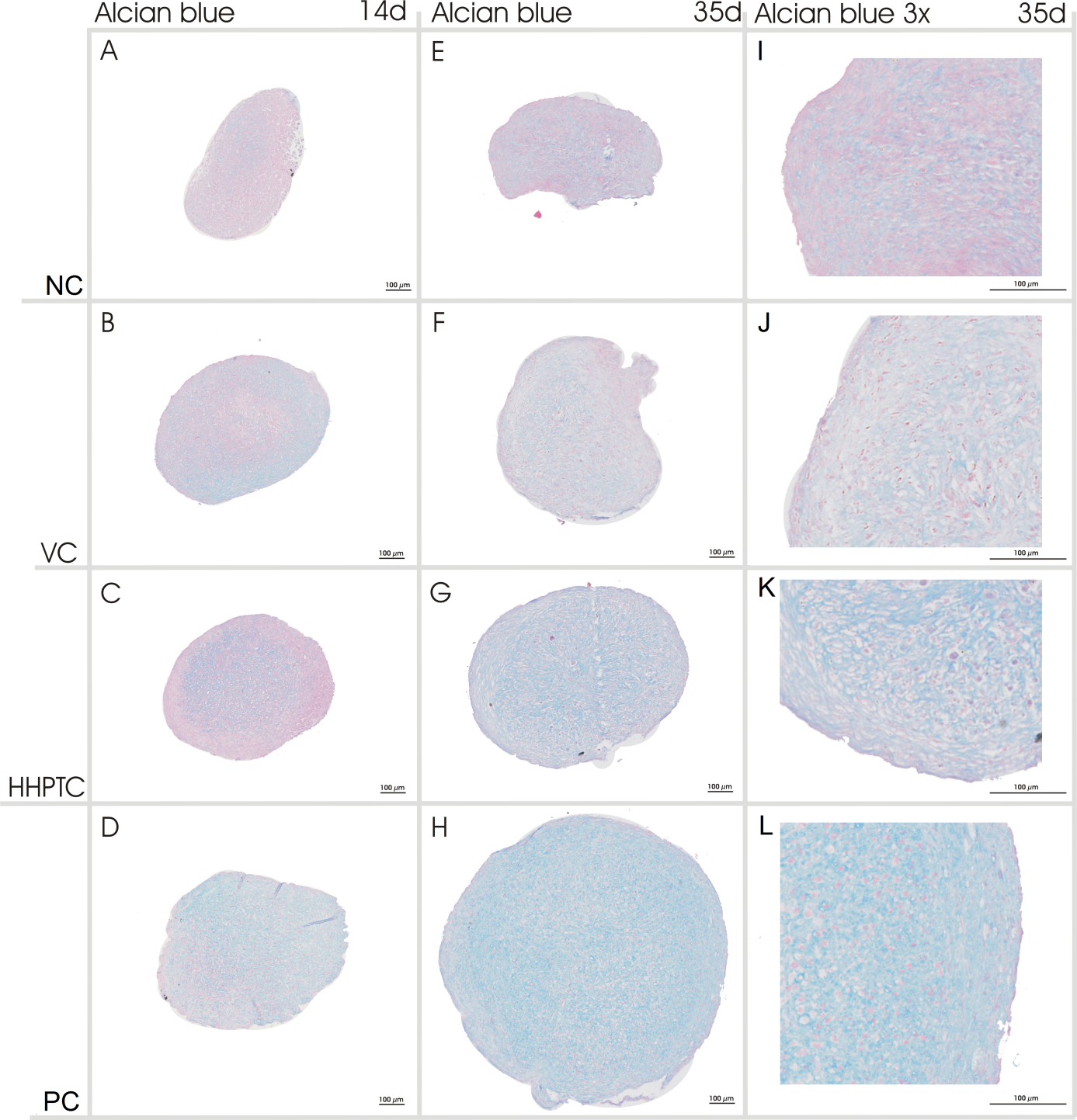


**Figure S3: Representative pictures of Alcian blue staining of chondrocytic spheroid cultures.** Histological staining of chondrocytic spheroid cultures was performed after 14 days (A-D) and 35 days (E-L) of cultivation with different medium groups: negative control (NC) (A,E,I), vital cartilage conditioned medium (VC) (B,F,J), high hydrostatic pressure treated cartilage conditioned medium (HHPTC) (C,G,K) and positive control (with chondrogenic growth factors, PC) (D,H,L). Three times magnified sections from stains E-H are depicted in pictures I-L. n=12, scale bar: 100 µm.


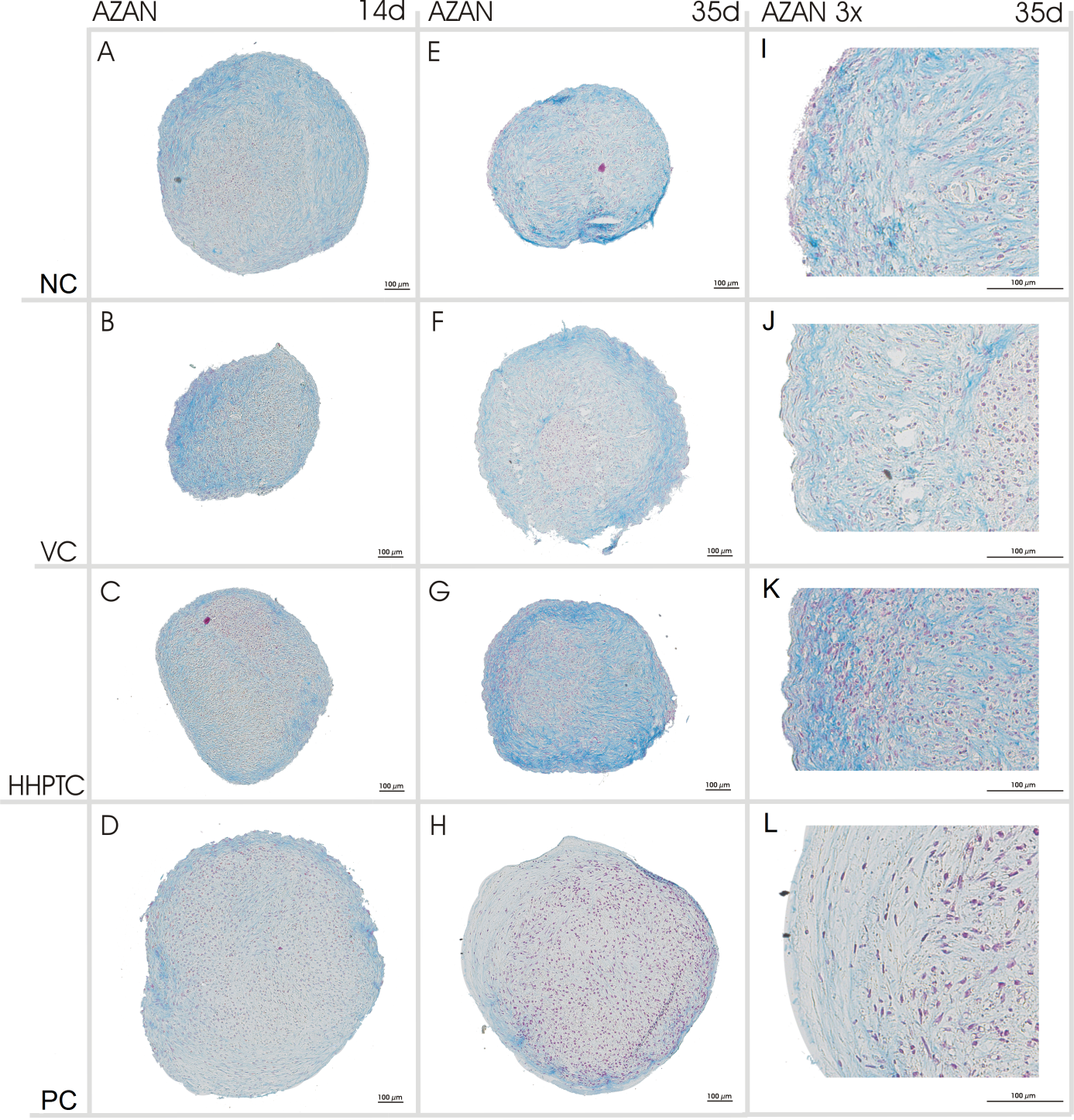


**Figure S4: Representative pictures of Heidenhain’s AZAN trichrome staining of chondrocytic spheroid cultures.** Histological staining of chondrocytic spheroid cultures was performed after 14 days (A-D) and 35 days (E-L) of cultivation with different medium groups: negative control (NC) (A,E,I), vital cartilage conditioned medium (VC) (B,F,J), high hydrostatic pressure treated cartilage conditioned medium (HHPTC) (C,G,K) and positive control (with chondrogenic growth factors, PC) (D,H,L). Three times magnified sections from stains E-H are depicted in pictures I-L. n=12, scale bar: 100 µm.
