# Supplementary figures and images for: Influence of Conditioned Media on the Re-Differentiation Capacity of Human Chondrocytes in 3D Spheroid Cultures
Source: J Clin Med. 2020 Aug 30;9(9):2798. doi: 10.3390/jcm9092798 (PMC7564315; doi:10.3390/jcm9092798)

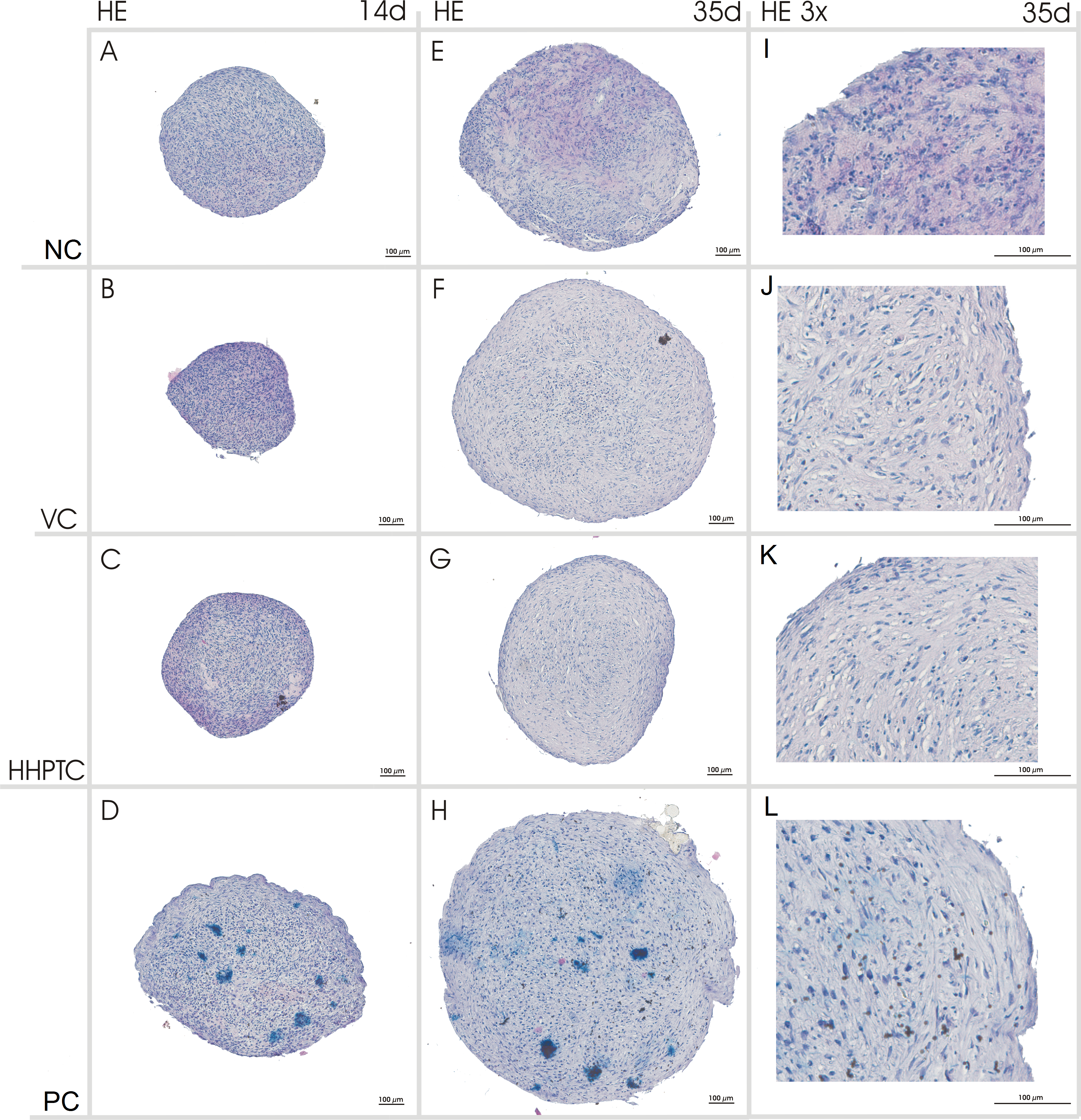

Supplement: Supplementary file 1 [file jcm-09-02798-s001.zip › Supplementary figures/Figure S2.tif]

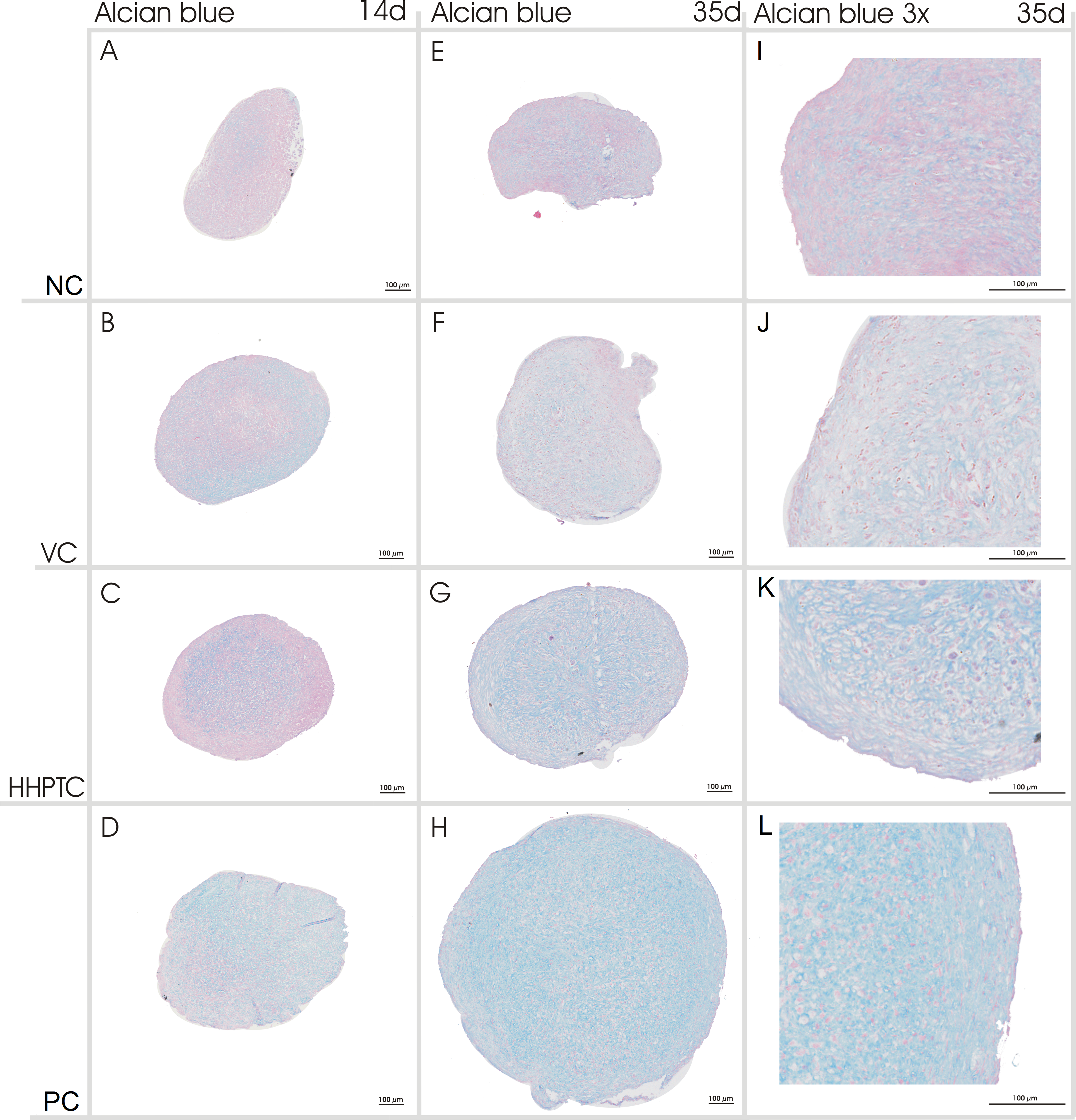

Supplement: Supplementary file 1 [file jcm-09-02798-s001.zip › Supplementary figures/Figure S3.tif]

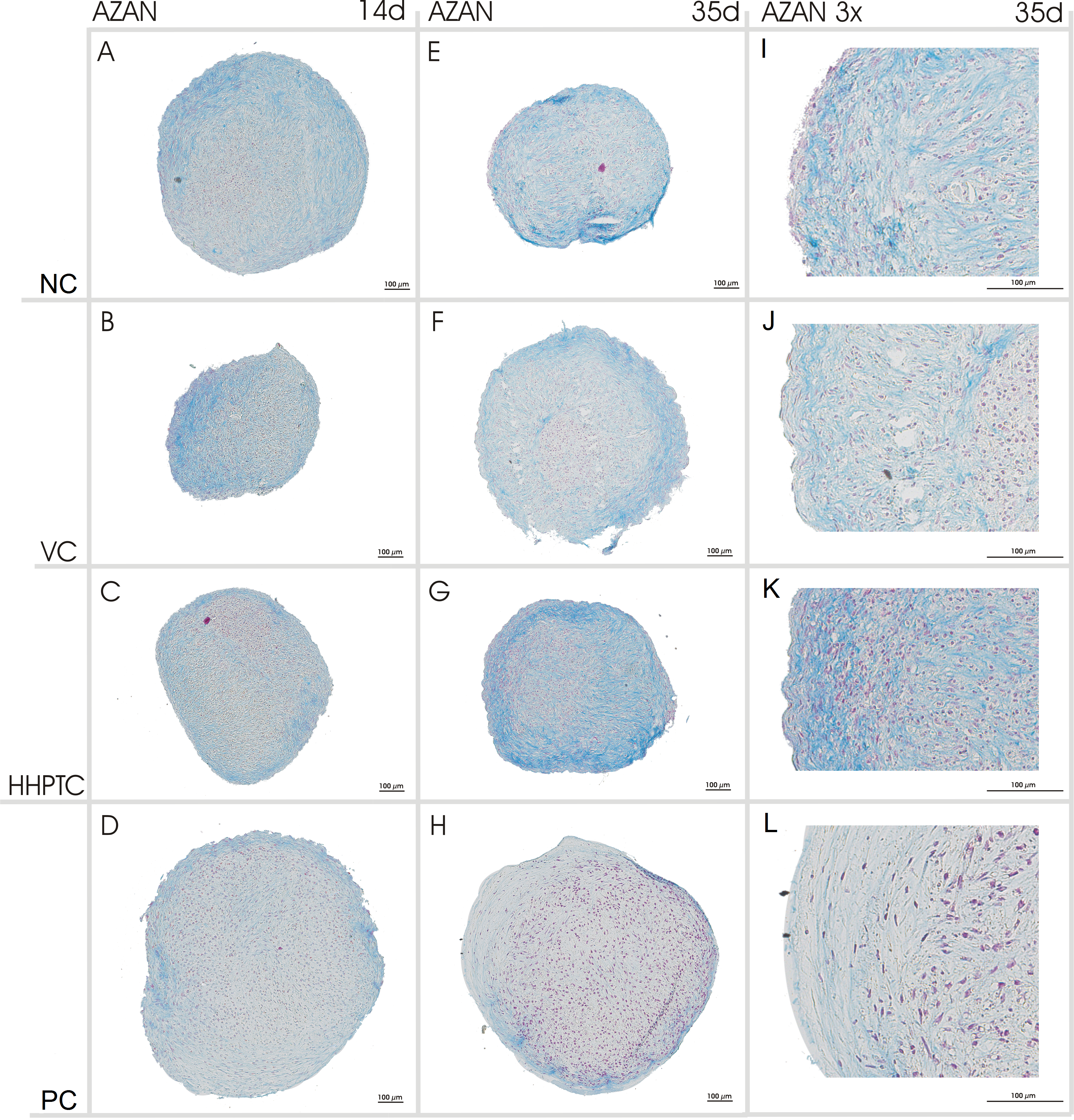

Supplement: Supplementary file 1 [file jcm-09-02798-s001.zip › Supplementary figures/Figure S4.tif]
